# Ice formation and solvent nanoconfinement in protein crystals

**DOI:** 10.1107/S2052252519001878

**Published:** 2019-03-13

**Authors:** David W. Moreau, Hakan Atakisi, Robert E. Thorne

**Affiliations:** a Physics Department, Cornell University, Ithaca, NY 14853, USA

**Keywords:** protein crystallography, nanoconfinement, ice, stacking disorder

## Abstract

Ice formation within protein crystals is a major obstacle to the cryocrystallographic study of protein structure, and has limited studies of how the structural ensemble of a protein evolves with temperature in the biophysically interesting range from ∼260 K to the protein–solvent glass transition near 200 K. Using protein crystals with solvent cavities as large as ∼70 Å, time-resolved X-ray diffraction was used to study the response of protein and internal solvent during rapid cooling. Solvent nanoconfinement suppresses freezing temperatures and ice-nucleation rates so that ice-free, low-mosaicity diffraction data can be reliably collected down to 200 K without the use of cryoprotectants. Hexagonal ice (I_h_) forms in external solvent, but internal crystal solvent forms stacking-disordered ice (I_sd_) with a near-random stacking of cubic and hexagonal planes. Analysis of powder diffraction from internal ice and single-crystal diffraction from the host protein structure shows that the maximum crystallizable solvent fraction decreases with decreasing crystal solvent-cavity size, and that an ∼6 Å thick layer of solvent adjacent to the protein surface cannot crystallize. These results establish protein crystals as excellent model systems for the study of nanoconfined solvent. By combining fast cooling, intense X-ray beams and fast X-ray detectors, complete structural data sets for high-value targets, including membrane proteins and large complexes, may be collected at ∼220–240 K that have much lower mosaicities and comparable *B* factors, and that may allow more confident identification of ligand binding than in current cryocrystallographic practice.

## Abstract 2

Nanoconfinement dramatically modifies the behaviour of solvent within protein crystals, allowing biophysical measurements in the presence of liquid solvent at temperatures down to ∼200 K. When internal ice forms it is stacking-disordered, consistent with nucleation within deeply supercooled solvent, and ice does not form in solvent within ∼5 Å of the protein surface.

## 1. Introduction   

Ice formation and its prevention are key issues in many areas of bioscience and biotechnology, including the cold-hardiness of microorganisms, animals and agriculturally relevant plants; the cryopreservation of cells, tissues and organs; the cold storage of proteins and biologics; and biomolecular and cellular structure determination using electrons and X-rays.

X-ray crystallography is our primary tool for probing biomolecular structure. In its early days, crystallography was performed using protein crystals at or near room temperature. Once synchrotron X-ray sources became widely available in the 1990s, data collection shifted to the near-exclusive use of cryogenically cooled crystals. Cryocooling to ∼100 K reduces the rate at which diffraction properties degrade with X-ray dose by a factor of ∼50, increasing the amount of data that can be collected per crystal, and reduces thermal motions, often increasing the resolution (Rupp, 2009 ▸; Pflugrath, 2015 ▸). In favourable cases, crystallography beamlines can now collect diffraction data sets sufficient for protein structure determination in less than one second.

However, proteins have complex, multi-tiered energy landscapes, and biologically relevant information is lost when crystals are cryocooled owing to the thermal freezing out of conformational motions (Halle, 2004 ▸; Fraser *et al.*, 2009 ▸; Keedy *et al.*, 2015 ▸) and owing to steric hindrances imposed by increased molecular packing densities – the same factors that are responsible for improved diffraction resolution. Only a handful of crystallographic studies have examined the temperature evolution of protein structure in the biophysically interesting regime down to the protein–solvent glass (or dynamical) transition near ∼200 K, where most nonharmonic motions are kinetically quenched and enzymatic activity ceases (Frauenfelder *et al.*, 1979 ▸; Tilton *et al.*, 1992 ▸; Teeter *et al.*, 2001 ▸). Recent studies (Keedy *et al.*, 2015 ▸) enabled by advances in electron-density interpretation and modelling (van den Bedem *et al.*, 2009 ▸; Fraser *et al.*, 2011 ▸; Lang *et al.*, 2014 ▸) have illustrated the unique potential of variable-temperature crystallography to provide all-atom, atomic resolution information about protein conformational ensembles, solvent structure and energy landscapes, and their connection to function.

A primary challenge in cryotemperature and especially variable-temperature crystallography is ice formation in crystal solvent, which disrupts the host protein crystal structure and leads to loss of ordered diffraction (Rupp, 2009 ▸; Pflugrath, 2015 ▸). Cryoprotectants such as glycerol, PEGs and alcohols are added to crystallization solutions or are used in post-crystallization soak solutions to suppress ice formation (Pflugrath, 2015 ▸). For data collection at 100 K, typical concentrations are 20–30%(*v*/*v*) (Pflugrath, 2015 ▸), increasing to 60%(*v*/*v*) or more for high-solvent-content [>80%(*v*/*v*)] crystals. For data collection between the homogeneous nucleation temperature of bulk water, *T*_h_ ≃ 235 K, and 180 K, crystals have been soaked in 75%(*v*/*v*) methanol (Tilton *et al.*, 1992 ▸). Cryo­protectants can stabilize proteins, but they can also perturb protein structure, degrade crystal diffraction and displace or be difficult to distinguish from weakly bound ligands in active sites (Pozharski *et al.*, 2013 ▸). Even when ice does not form, cryocooling to 100 K degrades long-range protein crystal order: mosaicities increase from <0.01° to 0.3° or more, leading to diffraction-peak overlap when the crystal unit cells are large. The challenges posed by cryocooling and ice formation are growing as the focus of structural studies shifts from smaller soluble proteins to membrane proteins, to large biomolecular complexes and to weakly packed, large-solvent-content crystal forms that are most likely to reveal native-like conformational ensembles and responses to optical, chemical or thermal perturbations.

Related challenges are encountered in the cryopreservation of protein solutions, cells and tissues (Fahy & Wowk, 2015 ▸). However, in protein crystals the solvent is nanoconfined within a periodic protein structure. Studies of water confined within nanoporous inorganic (primarily silica) matrices over the last two decades have shown that nanoconfinement dramatically modifies ice formation (Morishige & Kawano, 1999 ▸; Schreiber *et al.*, 2001 ▸; Jähnert *et al.*, 2008 ▸; Moore *et al.*, 2010 ▸; Suzuki, Steinhart *et al.*, 2015 ▸; Taschin *et al.*, 2015 ▸; Mascotto *et al.*, 2017 ▸). Deficiencies of the available matrices have complicated the interpretation of experiments, especially on non-equilibrium aspects such as nucleation, and have made studies with biophysically relevant solvent compositions difficult.

Here, we examine solvent behaviour and ice formation in protein crystals between 180 and 260 K, using data from over 400 crystals of three proteins with solvent cavities as large as ∼70 Å. We show that protein crystals enable new quantitative approaches to probing the effects of nanoconfinement on ice formation. Nanoconfinement strongly modifies the form and the formation of internal ice in protein crystals, and enables biophysical measurements of the conformational evolution and dynamics of proteins in the presence of liquid solvent at temperatures down to ∼200 K.

## 2. Methods   

### 2.1. Crystal growth, soaking and X-ray data collection   

Our studies focused on crystals of cubic apoferritin and tetragonal thaumatin, with additional measurements using tetragonal lysozyme (Supporting Information Section S1). All crystals were grown by the hanging-drop vapour-diffusion method in 24-well plates.

Crystals of equine spleen apoferritin (Sigma, catalog No. A-3641) were grown in hanging drops consisting of 2 µl of protein at 10 mg ml^−1^ in 0.1 *M* sodium acetate buffer pH 6.5 and 2 µl of a well solution consisting of 2%(*w*/*v*) CdSO_4_ and 15%(*w*/*v*) (NH_4_)_2_SO_4_ in the same buffer. Cubic crystals in space group *F*432 grew to dimensions of 300–500 µm within a week [Supplementary Fig. S1(*a*)].

Crystals of thaumatin (Sigma, catalog No. T7638) were grown in hanging drops comprised of equal volumes of protein at 40 mg ml^−1^ in 0.1 *M* sodium acetate buffer pH 6.5 and a well solution consisting of 14%(*w*/*v*) potassium/sodium tartrate in the same buffer. Tetragonal crystals in space group *P*4_1_2_1_2 grew to dimensions of 200–300 µm within one week [Supplementary Fig. S1(*b*)].

Crystals of lysozyme (Sigma, catalog No. L6876) were grown in hanging drops comprised of equal volumes of protein at 80 mg ml^−1^ in 0.1 *M* sodium acetate buffer pH 5.2 and a well solution consisting of 2.5%(*w*/*v*) NaCl in the same buffer. Tetragonal crystals in space group *P*4_3_2_1_2_1_ grew to dimensions of 300–800 µm. Crystals appeared within one week and stopped growing within four weeks.

Crystals were used as grown or else were cryoprotected by soaking for at least 5 min in glycerol solutions with concentrations of 10, 20 and 40%(*v*/*v*), which were obtained by adding glycerol to a solution with the same composition as the previously mentioned well solutions. Each crystal was transferred to a separate drop of NVH oil (Cargille) and manipulated until all external solvent was removed from its surface (Warkentin & Thorne, 2010*b* ▸). Crystals were mounted on microfabricated loops encapsulated in NVH oil to prevent dehydration during data collection and were stored in MicroRT tubes (MiTeGen) containing mother liquor or cryoprotectant solution for ∼1 h prior to data collection.

X-ray data were collected on station F1 at the Cornell High-Energy Synchrotron Source (CHESS) using a PILATUS 6M detector (Supporting Information Section S2). A cold nitrogen-gas stream programmed to the desired final sample temperature was directed at the crystal, but was initially blocked using a shutter. Each crystal was placed in the X-ray beam at room temperature, ten frames totalling 5° in rotation were collected to assess the crystal for damage or dehydration, and the crystal was then rotated back to its initial orientation. The gas stream was then unblocked and the collection of frames with 0.5° rotation and 0.1–0.2 s exposure per frame commenced (Supplementary Fig. S2).

### 2.2. Processing and modelling of protein structure diffraction   

Diffraction frames were indexed, integrated and scaled using *XDS* in segments of five frames (Supporting Information Section S3). Structural models were derived from frames starting after the unit cells reached a stable equilibrium and until the end of data collection. Molecular replacement and model refinement were performed using *PHENIX*, and the results were checked using *Coot* (Supporting Information Section S4). Protein and solvent volumes were then evaluated using the final refined models (Supporting Information Section S5). Refinement statistics for the 45 apoferritin and 53 thaumatin structures used in the analysis are given in the Supporting Information.

### 2.3. Processing and modelling of ice diffraction   

Diffraction frames from the detector were processed using Python scripts to remove protein Bragg scattering and background, and were azimuthally integrated (Supporting Information Section S6). The resulting intensity versus resolution plots were analysed by embedding the program *DIFFaX* (Treacy *et al.*, 1991 ▸), which calculates diffraction from samples containing stacking faults, in an optimization routine to determine the best-fit parameters for stacking-disordered ice formed of planes of hexagonal (I_h_) and cubic (I_c_) ice. These fits were compared with those obtained assuming a simple mixture of cubic and hexagonal crystallites (Supporting Information Section S7).

### 2.4. Estimating ice fractions in protein crystals   

Structure factors calculated from models of protein structure and ice diffraction were used to normalize protein structure and ice diffraction data collected from the same crystal in the same X-ray beam using the same detector, yielding the ratio of ice to protein crystal volume (Supporting Information Section S8). The ice volume was then compared with the solvent-cavity volume of the protein crystal at the ice observation temperature, with corrections applied based on estimates of the solvent fraction that exited the unit cell on cooling.

## 3. Results and discussion   

### 3.1. Solvent-content and solvent-cavity-size distributions in protein crystals   

**Figure 1:**
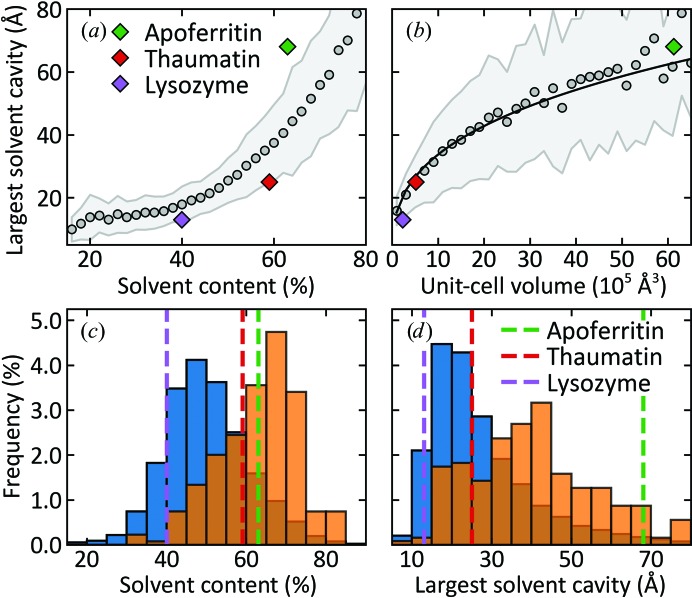
(*a*, *b*) Mean size and distribution of the largest solvent cavity within the unit cell versus solvent content and unit-cell volume obtained from 17 148 nonredundant protein structures in the PDB, excluding small peptides and viral proteins. Symbols indicate mean values and the shading indicates the region within one standard deviation of the maximum of the distribution. The solid line fitted in (*b*) has the form (cavity size) ∝ (cell volume)^1/3^, so cavity size scales with linear unit-cell dimension. (*c*, *d*) Histograms of PDB entry distributions versus solvent content and largest solvent cavity for soluble proteins (blue) and membrane proteins (orange). The corresponding values for cubic apoferritin, tetragonal thaumatin and tetragonal lysozyme crystals are marked in each frame.

Fig. 1 ▸ and Supplementary Fig. S4 show the size distribution and cumulative distribution, respectively, of the largest solvent cavity within the unit cell of a protein crystal versus solvent content and unit-cell volume for 17 146 nonredundant protein structures obtained from the Protein Data Bank (PDB; Supporting Information Section S9). Maximum solvent-cavity size, a primary determinant of ice formation during cooling, tends to increase with both solvent content and unit-cell volume. The diffraction resolution at 100 K degrades with increasing solvent content and solvent-channel size (Supplementary Fig. S5) owing to reduced constraints on atomic displacements from crystal-packing interactions and to the increased disorder caused by cryocooling. Membrane-protein crystals tend to have larger nonprotein volume fractions and larger solvent channels than soluble proteins [Figs. 1 ▸(*c*) and 1 ▸(*d*) and Supplementary Fig. S4], contributing to the difficulty in obtaining high-quality structural data sets from these crystals. For pure water in nanoporous silica and alumina, the effects of nanoconfinement on freezing and melting temperatures become pronounced for cavity sizes below ∼10 nm (Morishige & Kawano, 1999 ▸; Schreiber *et al.*, 2001 ▸; Jähnert *et al.*, 2008 ▸; Suzuki *et al.*, 2015 ▸; Taschin *et al.*, 2015 ▸; Mascotto *et al.*, 2017 ▸). Fig. 1 ▸ suggests that the effects of nanoconfinement on solvent behaviour should be pronounced in nearly all protein crystals.

**Figure 2:**
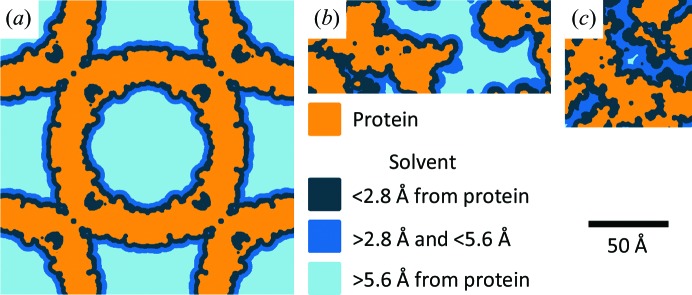
Solvent-cavity structure in (*a*) cubic apoferritin, (*b*) tetragonal thaumatin and (*c*) tetragonal lysozyme crystals at room temperature. The van der Waals surface of the protein is shown in orange. Solvent spaces within the first and second hydration shells are shown in dark blue and medium blue, respectively. Supplementary Movies S1–S6 provide detailed views of the protein and solvent-cavity structures.

We studied cubic apoferritin, tetragonal thaumatin and (with limited measurements) tetragonal lysozyme crystals, with Matthews-coefficient-derived solvent contents of 63, 59 and 42%(*v*/*v*) and maximum solvent-cavity sizes of 68, 25 and 13 Å, respectively, as indicated in Figs. 1 ▸ and 2 ▸. These maximum cavity sizes span the range of relevance in protein crystallography (Fig. 1 ▸), with the cavities of apoferritin being larger than those found in ∼98% of PDB entries (Supplementary Fig. S4). Although apoferritin and thaumatin have similar solvent contents, the fractions of solvent located beyond the first two hydration shells of the protein are very different (Fig. 2 ▸ and Supplementary Fig. S6).

**Figure 3:**
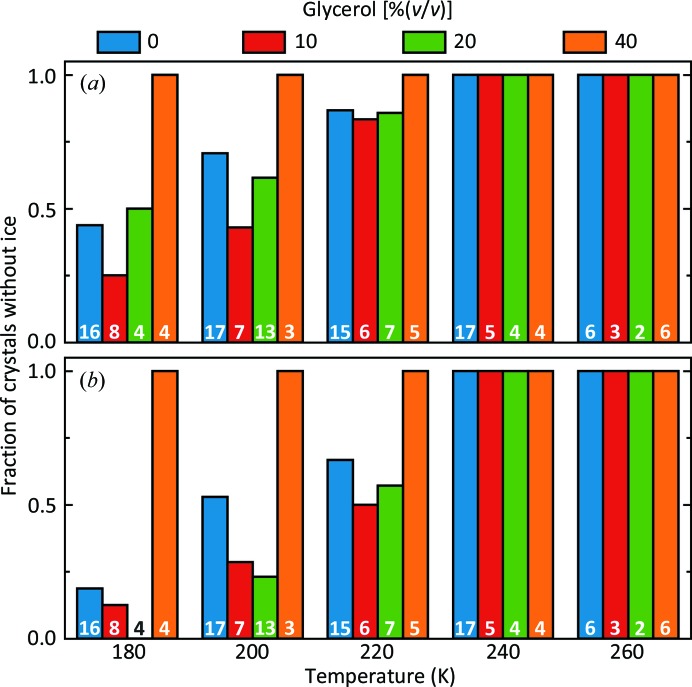
The fraction of apoferritin crystals at each temperature and glycerol concentration that remained free from ice diffraction for at least (*a*) 3 s and (*b*) 20 s after the unit cell reached its steady state or minimum value, excluding the ∼25% of crystals that formed hexagonal ice in external solvent. The numbers on each bar indicate the crystals examined for each condition. Data for thaumatin are given in Supplementary Fig. S7.

Time-resolved X-ray diffraction measurements were performed on apoferritin and thaumatin crystals soaked in solutions containing 10, 20 and 40%(*v*/*v*) glycerol or harvested as is [0%(*v*/*v*)] and then abruptly cooled (in <1 s) to temperatures between 180 and 260 K (Supporting Information Section S2). Fig. 3 ▸ shows the fraction of apoferritin crystals that remained free of internal ice and diffracted to high resolution for at least 3 s [Fig. 3 ▸(*a*)] and 20 s [Fig. 3 ▸(*b*)], times that are sufficient to collect complete structural data sets on high-brilliance synchrotron beamlines, after their unit cell reached its steady state or minimum value. No apoferritin crystals, regardless of the concentration of glycerol, showed internal ice formation at temperatures above 240 K. Ice eventually appeared below 240 K in crystals with lower glycerol concentrations. However, at all temperatures internal solvent within at least a substantial minority of these crystals could be maintained in a supercooled state for at least a few seconds. Ice first became detectable up to ∼20 s after the crystals reached their steady-state temperature. Similar results were obtained using thaumatin crystals (Supporting Information Section S10 and Supplementary Fig. S7). The salt concentrations present within the crystallization solutions and internal crystal solvent suppress bulk freezing temperatures by only a few degrees. When bulk solutions containing these salt concentrations are cooled below the freezing temperature, ice forms in ∼10 ms unless the temperature drops below the solvent glass-transition temperature *T*_g_ first (Supporting Information Section S11). Consequently, the suppression of ice formation in protein crystals with solvent cavities of up to ∼70 Å must be owing to nanoconfinement.

**Figure 4:**
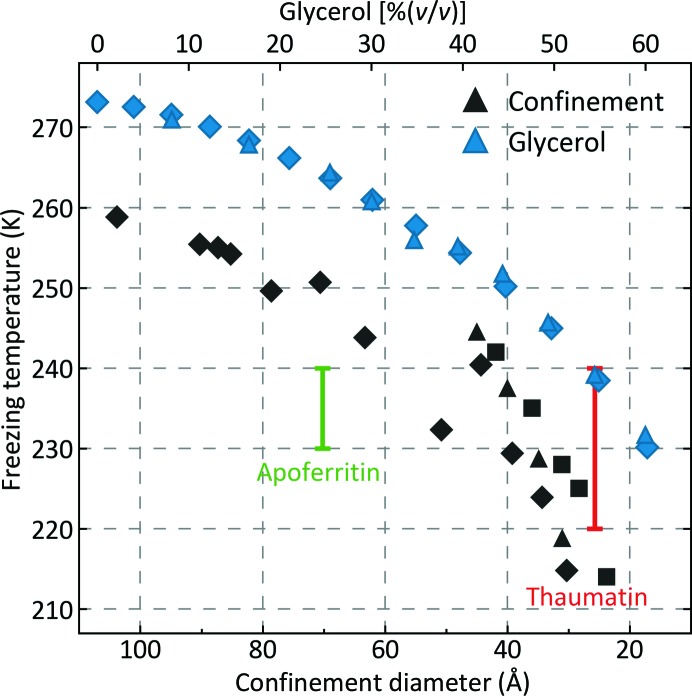
Blue points and upper scale: freezing temperature of bulk aqueous glycerol solutions versus glycerol concentration from Lane (1925 ▸) (triangles) and Segur (1946 ▸) (diamonds). Black points and lower scale: freezing temperature of pure water versus confinement diameter for confinement in cylindrical nanopores formed in silica from Findenegg *et al.* (2008 ▸) (triangles), Jähnert *et al.* (2008 ▸) (squares) and Kittaka *et al.* (2006 ▸) (diamonds). Confinement within ∼100 Å pores is as effective as adding 30–35%(*v*/*v*) glycerol in suppressing freezing temperatures.

As shown in Fig. 4 ▸, for pure water in nanoporous silica matrices the freezing temperature *T*_f_ decreases with decreasing pore diameter (Morishige & Kawano, 1999 ▸; Schreiber *et al.*, 2001 ▸; Jähnert *et al.*, 2008 ▸). For cylindrical pores, *T*_f_ is ∼250 and ∼223 K for diameters of 67 and 29 Å, respectively; no phase transition is observed for diameters below ∼20 Å (Jähnert *et al.*, 2008 ▸). The maximum temperatures at which ice forms in glycerol-free apoferritin and thaumatin crystals are comparable, based on their maximum solvent-cavity sizes, to these previous measurements.

Freezing-point suppression is an *equilibrium* effect of nanoconfinement. Long delays between cooling to below the freezing point and ice formation, and thus the persistence of metastable supercooled internal solvent, in glycerol-free apoferritin and thaumatin crystals at temperatures as low as 200 K indicate that solvent nanoconfinement within protein crystals dramatically modifies the *kinetics* of ice nucleation and growth. Reported nucleation rates between 193 and 215 K in supersonic nozzle-generated water nanodrops of diameters between 60 and 120 Å are of the order of 10^24^ cm^−3^ s^−1^ (Huang & Bartell, 1995 ▸; Manka *et al.*, 2012 ▸). Nucleation rates in micrometre-size drops near the bulk homogeneous nucleation temperature, *T*_h_ ≃ 235 K, are ∼10^9^ cm^−3^ s^−1^ (Murray *et al.*, 2010 ▸). These data, spanning 14 orders of magnitude in nucleation rate over Δ*T* ≃ 43 K, have been fitted with models of homogeneous nucleation (Murray *et al.*, 2010 ▸). Ice fractions of ∼1% are obtained when micrometre-size water drops are cooled at ∼10^5^–10^6^ K s^−1^ (Brüggeller & Mayer, 1980 ▸). Assuming 100 water molecules per nucleus (Huang & Bartell, 1995 ▸; Moore & Molinero, 2010 ▸) and that all ice is owing to nucleation (*i.e.* no post-nucleation growth) gives a nucleation rate of the order of 10^23^ cm^−3^ s^−1^, which is consistent with the peak temperature-dependent rate.

However, for water confined within ∼400 µm apoferritin crystals, the maximum solvent-cavity size of which is 68 Å, the solvent can remain as a metastable liquid on timescales of ∼10^0^–10^2^ s following cooling to 200–230 K. When ice eventually becomes detectable, its diffraction intensity saturates in as little as 0.2–0.4 s. Assuming that ice detection arises from a single nucleation event shortly before that detection, nucleation rates between 200 and 230 K are in the region of 10^6^ cm^−3^ s^−1^, which is ∼10^10^–10^17^ times smaller than in water nanodrops comparable in size to the solvent cavities in apoferritin. The most conservative assumptions, that ice is first detectable when the ice fraction reaches 2%, that nucleation occurs steadily until that threshold is reached, that each nucleus involves 100 water molecules and that nuclei do not grow, give a nucleation rate of ∼10^19^ cm^−3^ s^−1^, which is roughly four orders of magnitude smaller than in water nanodrops below 215 K. The grain sizes of internal ice in apoferritin crystals deduced from diffraction peak widths are in the range ∼200–800 Å, spanning many unit cells, so the actual nucleation rates are likely to lie between these limits. The dramatically reduced nucleation rates under nanoconfinement that we infer are qualitatively consistent with simulations (Li *et al.*, 2013 ▸), showing increasing suppression of nucleation in ∼30 Å drops relative to the bulk as temperatures increase above ∼210 K. However, they contrast with the much larger than bulk nucleation rates deduced from NMR experiments on nanoporous silica with 120 Å cavities (Mascotto *et al.*, 2017 ▸).

### 3.2. Protein crystal diffraction quality is maximized near *T* = 220 K in crystals with liquid solvent   

For ice-free apoferritin and thaumatin crystals, the Wilson *B* factors (a measure of short-range crystal disorder that is strongly correlated with resolution) decrease to a minimum near ∼220 K, and neither cooling to 100 K nor the use of glycerol provide clear improvements (Supplementary Fig. S8). For both proteins, the crystal mosaicities generally increase with decreasing temperature (Supplementary Fig. S9) and are smaller by factors of ∼2–6 at 220 K than at 100 K for all glycerol concentrations except 40%(*v*/*v*). Between 200 and 260 K, glycerol-free crystals of both proteins tend to have the lowest mosaicities.

### 3.3. Unit-cell contraction on cooling is not determined by internal solvent contraction   

**Figure 5:**
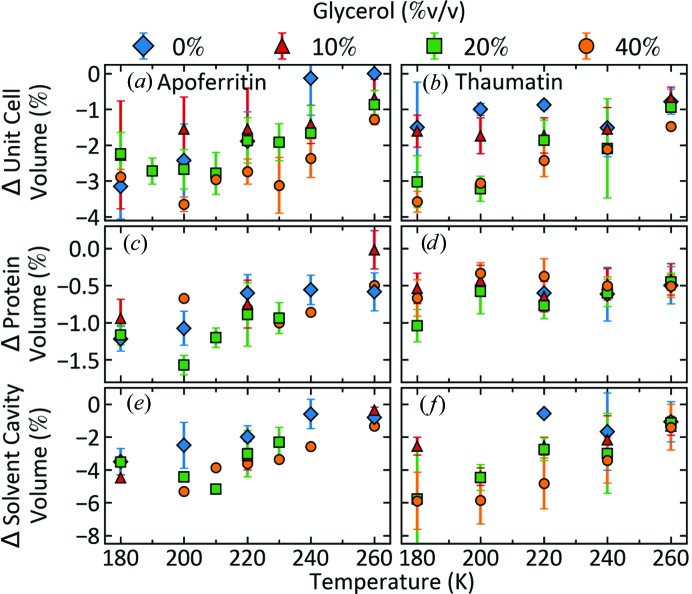
Changes in the unit-cell, solvent-cavity and protein volumes from their room-temperature values versus temperature for apoferritin (left column) and thaumatin (right column).

For ice-free apoferritin and thaumatin crystals at all glycerol concentrations, the unit-cell volumes measured ∼3–5 s after cooling contract monotonically between 300 and 180 K [Figs. 5 ▸(*a*) and 5 ▸(*b*)]. For both proteins, the contraction of the protein volume on cooling to 180 K is small (∼0.5–1%) and is nearly independent of glycerol concentration [Figs. 5 ▸(*c*) and 5 ▸(*d*)]. The solvent-cavity volume contraction is much larger and is only weakly dependent on the glycerol concentration [Figs. 5 ▸(*e*) and 5 ▸(*f*)].

As will be discussed in more detail elsewhere (Moreau *et al.*, 2019 ▸), internal solvent contraction on cooling cannot be the primary driver of these unit-cell and solvent-cavity volume contractions. A simple model for protein crystal volume changes on cooling from an initial (i) to a final (f) temperature is described by
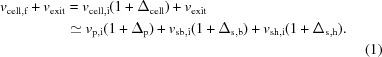
Here, *v*_cell,i_ and *v*_cell,f_ are the initial and final unit-cell volumes, respectively, *v*_exit_ is the amount of solvent that leaves the unit cell (owing to differential thermal contraction of the cell, protein and solvent; Juers & Matthews, 2001 ▸; Kriminski *et al.*, 2002 ▸), *v*_p,i_, *v*_sb,i_ and *v*_sh,i_ are the initial volumes of protein, bulk-like solvent and hydration (strongly perturbed) solvent, respectively, and Δ_cell_, Δ_p_, Δ_s,i_ and Δ_s,h_ are fractional changes in specific volumes on cooling. Pure bulk water expands by ∼6% on cooling from a room-temperature liquid to low-density amorphous (LDA) ice at 77 K, whereas a 40%(*v*/*v*) glycerol solution contracts by ∼5% (Tyree *et al.*, 2018 ▸). The largest fractional specific volume changes for both protein and solvent occur between 300 and 200 K. Assuming that *v*_exit_ = 0, that all solvent in apoferritin crystals has bulk-like volume contraction (*v*_sh,i_ = 0) and the same glycerol concentration as the soak solution, and that Δ_p_ does not depend on the glycerol concentration, the unit-cell volume at 100 K for 40%(*v*/*v*) glycerol crystals should be ∼7% smaller than for glycerol-free crystals. In fact, the unit-cell volumes of 40% glycerol apo­ferritin crystals are only 0.4 and 1.4% smaller than of glycerol-free crystals at 100 and 200 K relative to room temperature. Similar discrepancies between expected and measured unit-cell volumes are observed at temperatures between 180 and 260 K.

The most plausible explanation for these discrepancies is that *v*_exit_ is not zero (Juers & Matthews, 2001 ▸; Kriminski *et al.*, 2002 ▸; Juers *et al.*, 2018 ▸) and that on cooling a substantial amount of solvent exits (or enters) the ordered unit cells that contribute to Bragg diffraction (Supporting Information Section S5). With the most conservative assumptions, measured unit-cell contractions to 100 K for apoferritin give a *v*_exit_ of ∼9% of the room-temperature solvent-cavity volume for glycerol-free crystals and a *v*_exit_ of ∼−1.8% for crystals soaked in 40%(*v*/*v*) glycerol solutions (the negative sign implies that solvent must enter the unit cell). Similar results are obtained for thaumatin crystals. Table 1 ▸ gives estimates of the fraction of crystal solvent that exits the unit cells of glycerol-free crystals of apoferritin and thaumatin on cooling to temperatures between 180 and 260 K.

These results indicate that unit-cell contraction on cooling at rates of up to ∼1000 K s^−1^ is not primarily determined by internal solvent contraction or expansion. It is driven by the hydrated protein structure, by the reduction in protein entropy that accompanies side-chain ordering and the formation of additional crystal contacts (Juers & Matthews, 2001 ▸), and perhaps also by reduced hydration-layer solvent entropy.

### 3.4. Internal ice in protein crystals is stacking-disordered   

Ice diffraction is routinely observed in protein crystallo­graphy, and can arise both from internal solvent and from external solvent surrounding the crystal. An analysis of PDB-deposited data (generally, the best data obtained in a given set of experiments) found evidence of errors in protein crystal structure factors consistent with contamination by (and incomplete modelling of) ice in roughly 20% of entries (Thorn *et al.*, 2017 ▸). Ice diffraction from protein crystals has been discussed in terms of ideal hexagonal ice (I_h_), cubic ice (I_c_) and low-density amorphous ice (I_LDA_) patterns.

**Figure 6:**
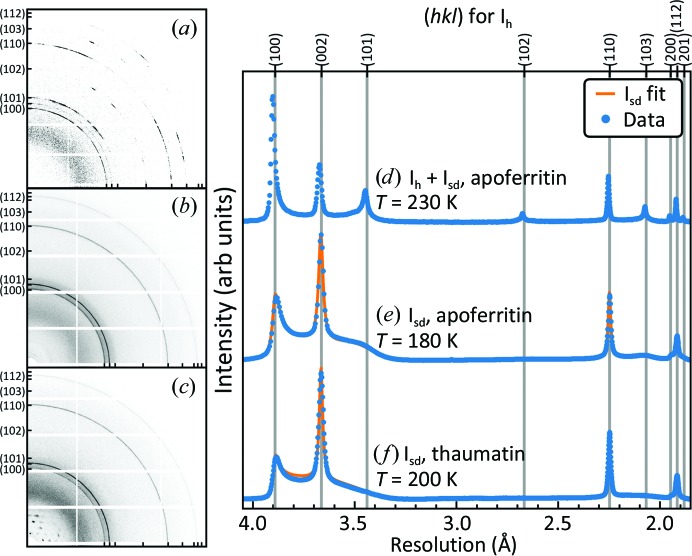
(*a*, *b*, *c*) Examples of detector images showing ice diffraction. (*a*) Mixture of hexagonal ice (I_h_) and stacking-disordered ice (I_sd_) in a glycerol-free apoferritin crystal at 230 K. (*b*) I_sd_ in a glycerol-free apoferritin crystal at 180 K. (*c*) I_sd_ in a glycerol-free thaumatin crystal at 200 K. (*d*, *e*, *f*) Dotted blue lines indicate azimuthally integrated and background-subtracted ice-ring diffraction profiles calculated from the detector images in (*a*), (*b*) and (*c*), respectively. The solid orange lines are best-fit profiles calculated using *DIFFaX*.

In the present experiments, we attempted to remove all external solvent. For glycerol concentrations of 0 and 10%(*v*/*v*) and temperatures from 180 to 230 K, roughly 70% of apoferritin crystals (70 of 98) eventually formed ice. Only three showed azimuthally integrated diffraction patterns consistent with pure I_h_. Another 26 crystals showed diffraction peaks at all expected I_h_ positions, but with peak shapes and intensities that were inconsistent both with pure I_h_ and any simple mixture of I_h_ and I_c_ grains [Figs. 6 ▸(*a*) and 6 ▸(*d*)]. For all 29 of these crystals, ice formed within ∼2 s of the start of cooling, suggesting that it nucleated during cooling, and many of the raw diffraction patterns had a component that was azimuthally ‘lumpy’ [Fig. 6 ▸(*a*)], indicating a small number of large ice grains. In some cases, residual frozen solvent was clearly visible on the crystal surface. We thus attribute the appearance of hexagonal ice as arising from nucleation in residual external solvent, not internal solvent.

By far the most common pattern of ice diffraction, which was observed in 41 of 98 apoferritin crystals and in 36 of 51 thaumatin crystals between 180 and 230 K, consisted of a strong but broadened peak near *d* = 3.7 Å, a weaker broad peak near *d* = 3.9 Å and broadened peaks near 2.2 and 1.9 Å [Figs. 6 ▸(*e*) and 6 ▸(*f*)]. The I_h_ peaks near 3.4 and 2.1 Å were absent or strongly suppressed, and a smooth shoulder was instead observed near the position of the 3.4 Å peak. Ice diffraction in this case was always uniform and isotropic [Figs. 6 ▸(*b*) and 6 ▸(*c*)], indicating a large number of small, randomly oriented grains. For the glycerol-free apoferritin crystals that developed these ice diffraction patterns, the mean time to ice formation was 6.4 s and the standard deviation was 8.3 s, suggesting delayed and stochastic nucleation. These systematics, together with estimated freezing temperatures for bulk-like internal solvent, indicate that the ice arises from nucleation within deeply supercooled internal solvent. Poor fits to the diffraction patterns obtained in refinement indicate that it is neither cubic nor hexagonal, nor a simple mixture of the two.

**Figure 7:**
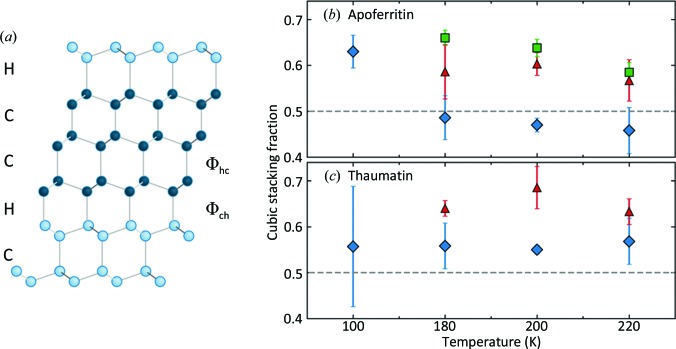
(*a*) Model of stacking-disordered ice (I_sd_; adapted from Malkin *et al.*, 2012 ▸) showing O atoms connected by hydrogen bonds. Along the vertical direction [normal to the (001) planes in I_h_ and to the (111) planes in I_c_], successive cubic ice planes are horizontally shifted, whereas successive hexagonal planes are mirror-reflected about a horizontal axis. On the left, C and H indicate pairs of planes that have cubic and hexagonal stacking, respectively. On the right, Φ_ch_ indicates the probability that a cubic stacking is followed by hexagonal stacking, and Φ_hc_ indicates the probability that a hexagonal stacking is followed by cubic stacking. (*b*, *c*) Cubic stacking fraction Φ_hc_/(Φ_hc_ + Φ_ch_) versus temperature for (*b*) apoferritin and (*c*) thaumatin crystals, determined from *DIFFaX* fits as in Figs. 6 ▸(*e*) and 6 ▸(*f*). The symbols indicate samples with different glycerol concentrations as in Fig. 5 ▸.

Similar diffraction patterns have been observed in experiments on ice formed on cooling in deeply supercooled water microdrops and nanodrops (Morishige & Uematsu, 2005 ▸; Malkin *et al.*, 2012 ▸, 2015 ▸; Kuhs *et al.*, 2012 ▸), on abrupt warming from a vitrified state and also in molecular-dynamics simulations (Moore & Molinero, 2011 ▸; Hudait *et al.*, 2016 ▸). They have been attributed to a disordered stacking of cubic and hexagonal ice planes along the hexagonal *c* direction [Fig. 7 ▸(*a*)].

In a simple model of this ‘stacking-disordered’ ice (I_sd_; Malkin *et al.*, 2015 ▸), the probability of a cubic plane being followed by a hexagonal plane is Φ_ch_ and that of a hexagonal plane being followed by a cubic plane is Φ_hc_ [Fig. 7 ▸(*a*)]. The solid lines in Figs. 6 ▸(*e*) and 6 ▸(*f*) are refined ice diffraction fits assuming this model (Supporting Information Section S7) calculated using the program *DIFFaX* (Treacy *et al.*, 1991 ▸). At all temperatures and all glycerol concentrations at which internal ice formed, the fits provide an excellent account of the observed diffraction. Fig. 7 ▸ shows that between 180 and 220 K the fraction of cubic layers is near 0.5, the value for purely random stacking, for glycerol-free crystals of both proteins and increases with glycerol concentration. The fit quality depends sensitively on the cubic stacking fraction (Malkin *et al.*, 2015 ▸), and the small crystal-to-crystal variance [indicated by the error bars in Figs. 7 ▸(*b*) and 7 ▸(*c*)] for the substantial number of crystals analysed (Supplementary Table S1) indicates that this stacking fraction is robust. A near-random stacking of cubic and hexagonal layers may result because the free energies of nucleating cubic and hexagonal ice planes on an ice-crystal surface are similar, so that competitive nucleation under conditions of deep supercooling leads to stacking that is dominated by kinetics rather than thermodynamics (Malkin *et al.*, 2015 ▸). The delayed formation of stacking-disordered ice within protein crystals is thus consistent with the existence of liquid, deeply supercooled internal solvent at temperatures down to ∼200 K.

Experiments on water nanoconfined within nanoporous silicas (MCM-41 and SBA-15; Baker *et al.*, 1997 ▸; Morishige & Uematsu, 2005 ▸) and ordered nanoporous aluminium oxide membranes (Suzuki, Duran *et al.*, 2015 ▸) observed evidence of stacking-disordered ice only when the pore diameters were larger than ∼200 and 350 Å, respectively; for smaller pores the internal ice was largely cubic. We observe disordered stacking with near-equal fractions of cubic and hexagonal planes in apoferritin and thaumatin crystals with maximum pore diameters of 68 and 25 Å, respectively. Accurate modelling of diffraction from internal ice in protein crystals, with its peak-dependent and asymmetric broadening, may improve the estimates of protein crystal structure factors and the accuracy of structural models.

### 3.5. Protein crystals enable novel quantitative estimates of crystallizable internal solvent fractions and perturbed interfacial layer thicknesses   

The structure and dynamics of water are locally perturbed by hydrogen bonding and other interactions with solutes, including proteins (Svergun *et al.*, 1998 ▸), and with interfaces, including the confining walls of nanoporous systems (Liu *et al.*, 2008 ▸; Erko *et al.*, 2012 ▸; Taschin *et al.*, 2015 ▸). Several experimental criteria (Bagchi, 2005 ▸) have been used to classify and quantify the fractions of locally perturbed and bulk-like water as a function of, for example, solute concentration or pore size. Perturbed layer thicknesses of ∼3–7 Å, in general agreement with simulations, are typically found, with substantial uncertainties arising from the models used to fit, for example, NMR lineshapes or calorimetric data. These compare with nominal thicknesses of the first and the first two hydration layers of 2.8 and 5.6 Å, respectively.

One metric of the perturbation of water is its ability to participate in a crystalline network (Sartor *et al.*, 1995 ▸; Rault *et al.*, 2003 ▸). Protein crystals enable a novel and highly quantitative approach to determining noncrystallizable solvent fractions (Supporting Information Section S8). Unlike in the most widely studied nanoconfining systems, the confining matrix provided by crystals of proteins such as apoferritin and thaumatin is nearly perfectly periodic and has excellent long-range order in all three dimensions, as indicated by sharp diffraction peaks with negligible strain broadening and mosaic broadening as small as 0.003° (comparable to that of silicon). Single-crystal diffraction from this matrix and powder diffraction from the internal ice confined within it can be recorded using the same X-ray beam and detector. Bragg diffraction from the protein matrix can be crystallographically modelled to determine its full atomic structure. Comparison of measured diffraction intensities with refined model structure factors yields a quantity related to the X-ray-illuminated volume of the crystal. Powder diffraction from internal ice, recorded after its intensity reaches a steady state, can be modelled using *DIFFaX*. Comparison of measured ice diffraction intensities with those from a refined ice model yields a quantity related to the total X-ray-illuminated volume of ice (Supporting Information Section S8). The ratio of these quantities from ice and protein crystal diffraction then gives the fraction of the X-ray-illuminated volume occupied by ice. Using the refined crystallographic models for the protein structure, the fraction of the unit cell occupied by solvent can be determined. This allows the fraction of internal solvent that forms ice to be determined with high accuracy.

As shown in Table 2 ▸ and Supplementary Table S2, the resulting maximum crystallized solvent-volume fractions at *T* = 180–220 K in glycerol-free crystals of apoferritin, thaumatin and lysozyme are ∼59, 35 and 17%, respectively. Accounting for possible solvent outflow from the unit cell owing to differential contraction of solvent and solvent cavities on cooling (Supporting Information Section S5, Table 1 ▸), these decrease to 47, 29 and 12%, respectively. The large difference in crystallizable solvent fraction between apoferritin and thaumatin crystals occurs despite only a 4% difference in their solvent contents. This difference may explain why apoferritin crystals lose nearly all ordered protein diffraction when ice forms, while thaumatin (and especially lysozyme) crystals continue to diffract to moderate resolution. The large crystallized solvent fractions for apoferritin and thaumatin confirm that the observed ice diffraction is from internal solvent: an external solvent volume with roughly 1/4 the volume of an ∼200–400 µm protein crystal would be visible to the naked eye and would immediately crystallize to form hexagonal ice on cooling.

By comparing these crystallizable solvent fractions with the cumulative distribution of solvent distances from the protein surface in each crystal (Supplementary Fig. S6), the thickness of the layer of noncrystallizable solvent adjacent to the protein surface can be determined. This thickness is of the order of 6 Å for all three proteins (Table 2 ▸), which is comparable to the thickness of the first two hydration shells, and is consistent with the approximate values estimated from studies of ice formation in hydrated protein powders (Sartor *et al.*, 1995 ▸) and porous inorganic glasses (Rault *et al.*, 2003 ▸).

## 4. Conclusions   

We have connected the behaviour of water and ice in protein crystals to results from previous studies of water in nanoporous inorganic matrices and in microdrops and nanodrops. Protein crystals have significant advantages over other nanoconfining systems. The ∼100 000 known protein crystal structures offer tremendous variety in pore size, pore geometry (including relatively simple geometries, as found in apoferritin) and chemical properties. A majority have excellent long-range order, so the full atomic structure of the confining matrix is known from crystallography and available for simulations. Ordered inorganic nanoporous matrices are typically synthesized as micrometre-size powders with large ratios of external surface area to volume, significant (5–10%) pore-size variations and substantial defect densities. Measurements on nanoconfined solvent require the use of packed powders, often filled from the vapour phase to minimize internal bubbles and overfilling, introducing substantial uncertainties and restricting study to pure water and other volatile liquids. Ice formation in solvent on the surface of individual grains is likely to corrupt the measurement of ice-nucleation rates.

In contrast, protein crystals are typically tens to hundreds of micrometres in size. Single crystals are sufficient for many measurements, and surface solvent can be optically detected and removed. The X-ray diffraction methods demonstrated here should allow ice nucleation and growth, grain sizes and crystallized solvent fractions to be tracked during and following cooling in single crystals with thermal response times of <100 ms. The composition of the internal solvent of a crystal can be changed by serial soaking in aqueous solutions containing salts, sugars, alcohols and polyols, including at concentrations (including nearly pure water) which cause crystal dissolution and/or protein unfolding, and often still retain excellent order, and the effects of, for example, the preferential hydration of protein surfaces and solute rejection by growing ice crystals on crystallizable solvent fractions can be determined. These features make protein crystals attractive model systems for studying the effects of confinement on ice formation, especially under biophysically relevant conditions.

Even in protein crystals with ∼70 Å solvent cavities, which are larger than those in ∼98% of current PDB depositions, nanoconfinement dramatically suppresses freezing temperatures (to ≤240 K) and ice-nucleation rates, with the latter allowing internal solvent to remain as a (supercooled) liquid for at least several seconds at temperatures between 200 and 240 K. By combining abrupt *in situ* cooling with intense synchrotron X-ray beams and fast X-ray detectors, complete structural data sets for high-value targets including membrane proteins and large complexes may be collected at ∼220 K that have much lower mosaicities and comparable *B* factors, and that may allow more confident identification of ligand binding than in current cryocrystallographic practice. This same crystal-based strategy of abrupt cooling and fast data collection before ice formation may enable a variety of temperature-dependent biophysical studies of protein structure, conformational ensembles and function, including at temperatures near *T*_h_ that are inaccessible when studying proteins in solution or *in vivo*.

## 5. Additional literature references   

The following references are cited in the supporting information for this article: Adams *et al.* (2010 ▸), Alcorn & Juers (2010 ▸), Amaya *et al.* (2017 ▸), Ashiotis *et al.* (2015 ▸), Atakisi *et al.* (2018 ▸), Bagchi (2005 ▸), Barbosa & Barbosa (2015 ▸), Bartell & Chushak (2003 ▸), Charron *et al.* (2002 ▸), Chen *et al.* (2010 ▸), Chukin *et al.* (2010 ▸), Crichton & Declercq (2010 ▸), Datta *et al.* (2001 ▸), Doster (2010 ▸), Douzou *et al.* (1975 ▸), Ebbinghaus *et al.* (2007 ▸), Emsley *et al.* (2010 ▸), Erko *et al.* (2012 ▸), Espinosa *et al.* (2014 ▸, 2016 ▸), Fenimore *et al.* (2004 ▸), Findenegg *et al.* (2008 ▸), Fokine & Urzhumtsev (2002 ▸), Fraser *et al.* (2009 ▸, 2011 ▸), Frauenfelder *et al.* (1979 ▸, 1987 ▸), Garman (2003 ▸), Garman & Schneider (1997 ▸), Gonzalez-Solveyra *et al.* (2011 ▸), Halle (2004 ▸), Hansen *et al.* (1996 ▸, 2008 ▸), Hare & Sorensen (1987 ▸), Holten & Anisimov (2012 ▸), Huang & Bartell (1995 ▸), Jahn *et al.* (2016 ▸), Jähnert *et al.* (2008 ▸), Juers & Matthews (2001 ▸, 2004*a* ▸,*b* ▸), Juers & Ruffin (2014 ▸), Kabsch (2010 ▸), Kantardjieff & Rupp (2003 ▸), Keedy *et al.* (2014 ▸, 2015 ▸), Knudsen *et al.* (2013 ▸), Kriminski *et al.* (2002 ▸), Kuffel & Zielkiewicz (2012 ▸), Kuhs *et al.* (1987 ▸, 2012 ▸), Lee *et al.* (2014 ▸), Li *et al.* (2013 ▸), Li & Nussinov (1998 ▸), Liu *et al.* (2010 ▸), Loerting *et al.* (2011 ▸), Malkin *et al.* (2012 ▸, 2015 ▸), Manka *et al.* (2012 ▸), Mascotto *et al.* (2017 ▸), Matthews (1974 ▸), Merzel & Smith (2002*a* ▸,*b* ▸), Merzel & Smith (2005 ▸), Miyatou *et al.* (2016 ▸), Moore & Molinero (2010 ▸, 2011 ▸), Moore *et al.* (2010 ▸), Morishige & Kawano (1999 ▸), Morishige & Nobuoka (1997 ▸), Morishige & Uematsu (2005 ▸), Murray *et al.* (2010), Nakasako (2004 ▸), Oliphant (2007 ▸), Parsegian *et al.* (2000 ▸), Persson *et al.* (2018 ▸), Petrov & Furó (2011 ▸), Pflugrath (2015 ▸), Rault *et al.* (2003 ▸), Riechers *et al.* (2013 ▸), Ringe & Petsko (2003 ▸), Rodgers (1994 ▸), Rupp (2009 ▸), Saraswathi *et al.* (2002 ▸), Schirò *et al.* (2011 ▸, 2015 ▸), Schmidt *et al.* (1995 ▸), Schreiber *et al.* (2001 ▸), Shen *et al.* (2016 ▸), Shimizu & Smith (2004 ▸), Sinibaldi *et al.* (2007 ▸), Suzuki, Steinhart *et al.* (2015 ▸), Svergun *et al.* (1998 ▸), Taschin *et al.* (2015 ▸), Teeter *et al.* (2001 ▸), Tilton *et al.* (1992 ▸), Timasheff (2002 ▸), Toby & Von Dreele (2013 ▸), Vekilov *et al.* (1996 ▸), Voss & Gerstein (2010 ▸), Waasmaier & Kirfel (1995 ▸), Wang *et al.* (2016 ▸), Warkentin *et al.* (2006 ▸, 2012 ▸, 2013 ▸), Warkentin & Thorne (2009 ▸, 2010*a* ▸), Warren (1990 ▸), Weik (2003 ▸), Weik *et al.* (2005 ▸) and Yao *et al.* (2017 ▸).

## Supplementary Material

Supplementary Movie S1. Animation of structure of cubic apoferritin. DOI: 10.1107/S2052252519001878/tj5020sup1.avi

Supplementary Movie S2. Animation of solvent cavities in cubic apoferritin crystals. DOI: 10.1107/S2052252519001878/tj5020sup2.mpg

Supplementary Movie S3. Animation of tetragonal thaumatin structure. DOI: 10.1107/S2052252519001878/tj5020sup3.avi

Supplementary Movie S4. Animation showing the solvent cavities in tetragonal thaumatin crystals. DOI: 10.1107/S2052252519001878/tj5020sup4.mp4

Supplementary Movie S5. Animation of tetragonal lysozyme structure. DOI: 10.1107/S2052252519001878/tj5020sup5.avi

Supplementary Movie S6. Animation of solvent cavities in tetragonal lysozyme. DOI: 10.1107/S2052252519001878/tj5020sup6.mpg

Spreadsheet S1. Refinement statistics for apoferritin data sets. DOI: 10.1107/S2052252519001878/tj5020sup7.xlsx

Spreadsheet S2. Refinement statistics for thaumatin crystals. DOI: 10.1107/S2052252519001878/tj5020sup8.xlsx

Supporting information, Supplementary Tables and Figures, and detailed captions to Supplementary Movies. DOI: 10.1107/S2052252519001878/tj5020sup9.pdf

## Figures and Tables

**Table 1:** Fractional changes in solvent-cavity volume and solvent volume (assuming bulk and interface-perturbed solvent contractions) on cooling from room temperature to each indicated temperature, and the fraction of solvent that must exit the unit cell, for apoferritin and thaumatin crystals calculated as described in Supporting Information Section S5 The volume fractions of the room-temperature unit cell occupied by solvent cavities, determined from structural models using the program *map_channels*, are 63.4 and 60.2% for apoferritin and thaumatin, respectively.

Fractional changes from room temperature to temperature *T*	180 K	200 K	220 K	240 K	260 K
Apoferritin–0% glycerol					
Change in solvent-cavity volume (%)	−3.5 ± 0.8	−2.5 ± 1.4	−2.0 ± 0.7	−0.6 ± 0.9	−0.8 ± 0.2
Change in solvent volume, ‘bulk’ (%)	6.5 ± 0.7	6.3 ± 0.7	3.7 ± 0.7	1.4 ± 0.7	0.2 ± 0.3
Change in solvent volume, ‘interface-perturbed’ (%)	5.3 ± 1.0	5.1 ± 1.0	3.0 ± 1.0	1.1 ± 1.0	0.2 ± 0.8
*f*_exit_, ‘bulk’ (%)	9.4 ± 0.8	8.3 ± 1.3	5.5 ± 0.6	1.9 ± 0.9	1.0 ± 0.2
*f*_exit_, ‘interface-perturbed’ (%)	8.4 ± 1.0	7.3 ± 1.5	4.9 ± 0.9	1.7 ± 1.1	1.0 ± 0.7
Thaumatin–0% glycerol					
Change in solvent-cavity volume (%)	−2.3 ± 0.8	−1.7 ± 0.8	−0.6 ± 0.2	−1.7 ± 2.4	−1.1 ± 1.2
Change in solvent volume, ‘bulk’ (%)	6.5 ± 0.2	6.3 ± 0.2	3.7 ± 0.2	1.4 ± 0.2	0.2 ± 0.2
Change in solvent volume, ‘interface-perturbed’ (%)	4.6 ± 0.8	4.4 ± 0.8	2.6 ± 0.7	1.0 ± 0.7	0.2 ± 0.7
*f*_exit_, ‘bulk’ (%)	8.3 ± 0.8	7.5 ± 0.8	4.1 ± 0.2	3.0 ± 2.3	1.3 ± 1.2
*f*_exit_, ‘interface-perturbed’ (%)	6.6 ± 1.0	5.9 ± 1.0	3.1 ± 0.7	2.6 ± 2.4	1.2 ± 1.4

**Table 2:** Estimates of the maximum fraction of the solvent-cavity space occupied by ice in glycerol-free crystals of apoferritin, thaumatin and lysozyme at temperatures between 180 and 220 K, as described in Supplementary Section S8

	Fraction of internal solvent that forms ice		
	Apoferritin	Thaumatin	Lysozyme
Fraction of solvent exiting the unit cell (%)			
*f*_exit_ = 0	59 ± 13	35 ± 6	17 ± 5
*f*_exit_, ‘bulk’	45 ± 13	25 ± 6	8 ± 6
*f*_exit_, ‘perturbed’	47 ± 13	29 ± 6	12 ± 6
Fraction of solvent >2.8 Å from protein (%)	83	70	29
Fraction of solvent >5.6 Å from protein (%)	55	31	0.5
Estimated thickness of unfreezable layer (Å)	6.6 ± 1.8	6.9 ± 0.9	5.1 ± 1.4
