# Supplementary material for: Ice formation and solvent nanoconfinement in protein crystals
Source: IUCrJ. 2019 Mar 13;6(Pt 3):346–56. doi: 10.1107/S2052252519001878 (PMC6503922; doi:10.1107/S2052252519001878)
Supplement: Supplementary file 9 [file m-06-00346-sup9.pdf]

IUCrJ

Volume 6 (2019)

Supporting information for article:

Ice formation and solvent nanoconfinement in protein crystals

David W. Moreau, Hakan Atakisi and Robert E. Thorne

S1. Properties of apoferritin, thaumatin, and lysozyme crystals

Our studies focused on crystals of cubic apoferritin and tetragonal thaumatin, with additional measurements on crystals of tetragonal lysozyme.

Apoferritin is comprised of twenty-four ferritin monomers, forming a 476 kDa complex having a nearly spherical cavity of diameter 68 Å for storage of iron; in the *apo* form the cavity is filled with solvent (Fig. 2(a) and Movie S1).

The solvent cavities in cubic apoferritin crystals have a face-centered cubic arrangement. These cavities are shown in Movie S2, where the unit cell origin has been shifted to place an apoferritin molecule in the center of the unit cell (making it appear as a body centered cubic arrangement). These large solvent cavities, their relatively simple geometry, and the resulting large fraction of bulk-like solvent make cubic apoferritin crystals excellent model systems for studies of ice formation under nanoconfinement. These large cavities also in part explain why previous crystallographic studies at T=100 K required relatively large cryoprotectant concentrations to prevent ice formation. Although the solvent cavities appear isolated from one another, when crystals are soaked in solutions containing iron, glycerol, or other small solutes/cryoprotectants, the non-aqueous components diffuse freely into or out of the cavities.

In tetragonal thaumatin crystals (Fig. 2(b) and Movies S3 and S4), the solvent is largely contained within twisted channels having a maximum diameter of 25 Å, near the peak of the solvent cavity size distribution in Fig. 1(d).

As shown in Fig. S1(b), thaumatin crystals grew as octahedra with flat faces. Complete removal of external solvent from the crystal facets was straightforward, and none of the crystals studied here showed evidence of external ice in diffraction. Apoferritin crystals (Fig. S1(a)) had more complex habits that made external solvent removal more difficult, and roughly 25% of glycerol-free crystals showed diffraction from external ice.

Fig. S6 shows the distribution of distances between solvent atoms and the nearest protein surface in apoferritin, thaumatin, and lysozyme crystals, calculated using map_channels as discussed in Sec. S9. In lysozyme nearly all solvent molecules are within 6 Å of the protein surface (i.e., roughly within the first two hydration shells), whereas in apoferritin only ~50% of solvent molecules are that close.

S2. X-ray data collection

Time-dependent X-ray diffraction data was collected using the F1 beamline at the Cornell High-Energy Synchrotron Source (CHESS) using the experimental configuration shown in Fig. S2. The F1 station uses a horizontally focusing monochromator using a single bent triangular Si(111) crystal. The monochromator Bragg angle for the (111) reflection of silicon at this energy is 9.0°. Samples were

illuminated using a Gaussian beam with a 65 μm FWHM and a divergence of 0.03°. The photon flux of 2.2×10^9 ph/s and photon energy of 12.7 keV gave dose rates of ~2 kGy/s. Diffraction patterns were recorded by a Pilatus 6M detector using a frame rate of 5 Hz.

Sample temperature was controlled using a nitrogen gas stream with a flow rate of 5 L/min generated by an Oxford Cryosystems Cryostream 700 nitrogen gas cryocooler. The gas stream's temperature was varied between 100 K and 260 K using the cryocooler's internal heater, and was monitored using both the cryocooler's internal temperature sensor and using a thermocouple that was periodically placed at the sample position. For room temperature measurements, the cryocooler head was retracted and the gas stream was blocked using an air blade shutter. To commence cooling, the cryocooler head was extended and the air blade shutter was turned off.

Fig. S3 shows the temperature at the sample position recorded using a 250 μm bead thermocouple, when the air blade shutter was turned off at t=0 and the gas stream temperature was set to 200 K. The maximum cooling rate is ~300 K/s. Actual sample cooling times, deduced from the thermal contraction of the unit cell, varied somewhat with crystal size and amount of surrounding oil but were in the range of 0.5-2 s.

Diffraction data were acquired before, during, and after sample cooling as follows. With the cryostream retracted and blocked, a sample in its MicroRT tube was manually placed on the beamline goniometer stage, the crystal was allowed to settle in the oil, and then the crystal was translated to the position of the X-ray beam. The MicroRT tube was then removed, and automated data collection initiated using the beamline's ADX software. In a typical experiment, an initial set of 10 frames (0.5° of sample rotation and 0.2 s exposure time per frame, 5° rotation and 2 s total per exposure), was collected at room temperature. The crystal was then returned to its initial orientation, the cryostream was extended and unblocked, and a single set of 200 frames (0.5°, 0.1 or 0.2 s exposure time per frame, 100° rotation and 20-40 s total exposure) acquired. With a dose rate of ~2 kGy/s, total doses ranged from ~40 to ~100 kGy, much less than the half dose at all temperatures studied, so changes in diffraction properties with time were dominated by effects other than radiation damage.

A set of early experiments used a slightly different protocol. An initial set of room temperature frames were collected as described above. A set of 20 frames was collected, the crystal was rotated back to its starting orientation, and 8 additional sets of 5 frames each, recorded using the same starting orientation, were collected. This protocol was abandoned for subsequent experiments because ice could form while the samples were being returned to the initial orientation for the start of each set, and so diffraction frames recording ice formation would not be recorded. Most of the data collected by this protocol was from apoferritin crystals with glycerol concentrations of 20% and 40% v/v, for which ice formation was rare in any case.

Using this protocol, diffraction data was collected from crystals cooled to temperatures between 180 K and 260K. Cooling of each crystal was monitored using the time-dependent lattice parameters

deduced from successive diffraction frames. Additional T=100 K data sets were collected using crystals that were plunge cooled in liquid nitrogen.

A total of 261 crystals of apoferritin, 168 crystals of thaumatin, and 4 crystals of lysozyme were examined at CHESS on seven different dates between November 2015 and March 2018. Measurement of a large number of samples was essential to drawing robust conclusions, because ice formation was studied versus both temperature and glycerol concentration; ice formation is stochastic; complete removal of external solvent was not always achieved, so external ice sometimes formed; the sample response depended to some extent on cooling time, which varied with crystal size and volume of surrounding oil; and because some samples developed cracks and other defects during post-growth handling that affected their response.

S3. Processing of protein lattice diffraction

Diffraction frames were indexed, integrated, and scaled using *XDS* (Kabsch, 2010), with an input file acquired from the MacCHESS website and modified for use with a Pilatus 6M detector. Diffraction was generally strong to beyond 3.0Å for apoferritin and to beyond 1.7Å for thaumatin, and mosaicities were generally low and peaks were well-separated, so processing was usually straightforward. Data sets were processed in segments of 5 frames. A segment's refined outputs of unit cell, beam center, and sample to detector distance were used as inputs in the XDS.INP file when processing subsequent segments. As a check on these results, data were also processed using *XDS* in segments of 2 and 10 frames, and using *HKL2000*). The use of 5 frame segments balanced time resolution with variance in parameter estimation.

Unit cell values were taken directly from *XDS*'s CORRECT.LP output file.

Wilson *B* factors were estimated as half the slope of a linear fit to the natural log of the Bragg peak intensities (obtained using *phenix.merging_*stats (Adams *et al.*, 2010)) vs. $(\sin\theta/\lambda)^2$. Bragg peaks having resolutions better than (i.e., numerically smaller than) 4 Å were used to determine *B* factors.

The refined beam divergence and crystal mosaicities, calculated using *XDS* as the standard deviation of Gaussians, were 0.015° and 0.045°, respectively. This latter value was a "floor" imposed by *XDS* that did not reflect actual crystal mosaicities. Diffraction frames were also indexed, integrated and scaled using *HKL2000*, which had a mosaicity "floor" close to the refined beam divergence. Mosaicity values reported here were calculated using values from the *HKL2000* *.x integration files, converted from full width at half maximum to standard deviation to allow comparison with *XDS* results.

Figs. S8 and S9 show Wilson B factors and mosaicities, respectively, for apoferritin and thaumatin crystals vs temperature and glycerol concentration.

S4. Protein structure modelling and refinement

Data frames were indexed and scaled using *XDS*, and molecular replacement and model refinement were performed using *PHENIX* (Adams *et al.*, 2010). PDB entries 3F32 and 3ZEJ were used as initial molecular replacement models for apoferritin and thaumatin, respectively. An initial refinement cycle using phenix.refine was performed, with simulated annealing, rigid body, real-space, xyz coordinates, and individual B-factor options. In *Coot* (Emsley *et al.*, 2010), these models were checked for large peaks in the difference maps, Ramachandran outliers, rotamer outliers, and regions of poor geometry. For apoferritin, cadmium ions and residues at a partially disordered loop, Gly155 and Ser156, were added into the model. For thaumatin, a well-ordered tartrate molecule was added. A second round of refinement was performed to identify and add ordered solvent. These models were checked residue by residue for errors and alternative conformations added. A third round of refinement including occupancies and B factors for alternative conformers and target weights was performed. Final model validation was performed using *MolProbity* in the *PHENIX* software package (Chen *et al.*, 2010). Structures with less than 98% completeness and resolutions worse than 2.1Å and 1.8 Å for apoferritin and thaumatin were excluded from analysis. Most structures were determined using a single crystal, but when that was not possible, data from several crystals having the same glycerol concentration and data collection temperature was merged using *XSCALE*. Refinement statistics for 45 apoferritin data sets and 53 thaumatin data sets are given in the Supporting Information.

S5. Protein and solvent volume calculations

Protein volumes within the unit cell $v_{protein}(T)$ were calculated as the volume enclosed by the solvent excluded surface (SES), using a custom "ball rolling" algorithm very similar to that implemented by the program 3vee (Voss & Gerstein, 2010). In 3vee, the SES is calculated by “rolling” a fixed-diameter probe over the surface of a voxelized 3D model of the protein with atoms assigned Van der Waals radii according to Ref. (Li & Nussinov, 1998). Our program was designed to also find the volume for multiple copies of the protein extended to fill the unit cell, accounting for crystal contacts and periodicity. Additionally, it uses probe radii of 1.4 Å and 1.7 Å for polar and apolar atoms, respectively (Li & Nussinov, 1998). Grid sizes were 0.15 Å for apoferritin and 0.10 Å for thaumatin and lysozyme.

Solvent cavity volumes $v_{cavity}(T)$ were determined by subtracting the protein volumes $v_p(T)$ from the unit cell volumes $v_{cell}(T)$. At room temperature, all crystals were highly ordered with very small mosaicities. Consequently, the solvent volume within a crystal could be assumed to be fully contained within the solvent cavities, and the crystal's solvent volume fraction was equal to the volume fraction of the unit cell occupied by cavities,

$$f_{solvent}(300\text{ K}) = \frac{v_{cavity}(300\text{ K})}{v_{cell}(300\text{ K})} \quad . \tag{S1}$$

At temperatures of 240 K and below, crystals generally had larger mosaicities indicating significant lattice-scale disorder. The total solvent volume within a crystal (per unit cell) $v_{solvent}(T)$ was assumed to be equal to the sum of the solvent cavity volume $v_{cavity}(T)$ (determined from crystallographic analysis as above) and the volume of solvent that exited the unit cell during cooling $v_{exit}(T)$. The exiting solvent is assumed to accumulate in disordered crystal regions that did not contribute to ordered Bragg diffraction.

As a first approximation, the total solvent volume at temperature *T* can be obtained from the room temperature solvent volume by scaling this volume by the thermal expansion of bulk solvent having the same composition,

$$v_{solvent}(T) = v_{solvent}(300\text{ K})\left(1+\Delta_{solvent,bulk}(T)\right), \tag{S2}$$

where $\Delta_{solvent,bulk}(T)$ gives the solvent volume expansion or contraction relative to room temperature. The volume fraction of solvent that exits the unit cells is then

$$f_{exit}(T) = \frac{v_{solvent}(T) - v_{cavity}(T)}{v_{solvent}(T)} \, . \tag{S3}$$

The thermal expansions of water and aqueous glycerol solutions can be obtained from density measurements (Hare & Sorensen, 1987; Loerting *et al.*, 2011; Shen *et al.*, 2016), and by extrapolations based on measurements and MD simulations (Holten & Anisimov, 2012; Jahn *et al.*, 2016) at temperatures where measurements are not available. The effects of salts at concentrations present within crystal solvent on expansion behaviour should be modest.

However, this "bulk" solvent approximation assumes that the internal solvent has the same expansion behaviour and composition as the crystallization or soaking solution. Disruption of water's hydrogen bonding network in the immediate vicinity of the protein surface increases its density there (Svergun *et al.*, 1998; Merzel & Smith, 2005; Kuffel & Zielkiewicz, 2012; Barbosa & Barbosa, 2015; Persson *et al.*, 2018); the increase from bulk density is estimated to be 6% and 1%, respectively, for the first and second hydration shells (Persson *et al.*, 2018). Glycerol concentrations in solvent cavities are likely smaller due to preferential hydration of protein surfaces (Timasheff, 2002; Parsegian *et al.*, 2000; Shimizu & Smith, 2004; Sinibaldi *et al.*, 2007; Datta *et al.*, 2001; Saraswathi *et al.*, 2002; Charron *et al.*, 2002). To give a rough estimate of uncertainties due errors in solvent density, the total solvent volume at temperature *T* was estimated assuming that solvent in the first hydration shell remained at a constant density on cooling, and that the remaining solvent had bulk-like composition and thermal expansion behaviour. Defining $f_{1^{st}\text{ shell}}$ as the fraction of solvent in the first hydration shell, this "interface-perturbed" solvent volume estimate,

$$v_{solvent}(T) = \left(1 - f_{1^{st}\,shell}\right) \cdot v_{solvent}(300\text{ K})\left(1 + \Delta_{solvent,bulk}(T)\right) + f_{1^{st}\,shell} \cdot v_{solvent}(300\text{ K}), \quad \text{(S4)}$$

was then used in Eq. S3 to estimate the fraction of solvent that exited the crystal.

Table 1 lists the room temperature solvent volume fraction $f_{solvent}(300\text{ K})$, the fractional solvent cavity volume change relative to room temperature at temperature *T*, and "bulk" and "interface-perturbed" estimates of the fractional solvent volume change and exiting solvent fraction $f_{exit}(T)$ for apoferritin and thaumatin crystals.

S6. Determining ice diffraction intensities

Diffraction images were loaded into python using the package *FabIO* (Knudsen *et al.*, 2013), which allowed the data to be handled as a numpy array, and were azimuthally averaged using the *pyFAI* integration package (Ashiotis *et al.*, 2015). Ice formation usually caused major loss of protein diffraction resolution, so protein lattice Bragg diffraction at the 2θ positions of the ice rings was generally negligible. When Bragg peaks persisted, they were removed using the *separate* function of *pyFAI*'s azimuthal Integrator module. *pyFAI* reduced the 2-dimensional Bragg-peak-masked diffraction data from the detector (counts at pixel coordinates *x*, *y*) to 1-dimensional diffraction data (average counts in a bin of angular width $\Delta(2\theta)$) versus angle 2θ or resolution $d = \lambda / 2\sin(\theta)$, correcting for beam polarization (90%, in the horizontal direction) and for variations in solid angle recorded by each pixel with 2θ. Accurate powder diffraction analysis relies on the sample being a 'perfect powder', which should generate diffraction rings that are smooth and symmetric about the beam polarization direction. Raw diffraction images were carefully inspected, and ice ring diffraction intensity in each ring versus azimuthal angle ϕ (determined as the angle between the *x* and *y* detector coordinates relative to the direct beam position) was plotted in 1° increments. Only samples for which these plots were uniform and symmetric in ϕ were used in quantitative analysis and fitting.

The non-Bragg X-ray background had contributions from the sample — NVH oil scattering, protein diffuse scattering, scattering from unfrozen water or low-density amorphous ice — and from the experimental set-up (including air scattering). The background was determined by evaluating a 10th order polynomial fit to the difference $I_{bg}(q) = I_{\exp}(q) - I_{DIFFaX}(q)$ between the *DIFFaX* models described below and our experimental diffraction patterns (similar to the method used in the powder diffraction analysis program *GSAS-II* (Toby & Von Dreele, 2013)), and then optimizing until the *DIFFaX* model and background converged. The resulting background fits were slowly varying curves (on the 2θ or resolution scale of the ice diffraction peaks) with a consistent shape between samples that captured the diffracted intensities from water and NVH oil.

S7. Modeling ice diffraction

Observed azimuthally integrated 1D ice diffraction patterns versus 2θ or resolution d for apoferritin and thaumatin crystals varied with temperature and glycerol concentration. The ice diffraction patterns were of four basic types: (a) azimuthally uniform and broad diffraction consistent with low-density amorphous ice I_{LDA}; (b) azimuthally lumpy, radially very narrow diffraction with intensities roughly consistent with I_h; (c) azimuthally lumpy diffraction with an azimuthally uniform component, the latter component consisting of a mix of narrow and broad peaks; and (d) azimuthally uniform patterns showing a mix of narrow peaks and broad, asymmetric peaks characteristic of stacking disordered ice I_{sd} (a disordered stacking of (001) planes of I_h and (111) planes of I_c). Of these, patterns of type (d) were by far the most common at temperatures between 180 K and 240 K.

Azimuthally uniform ice diffraction patterns were analyzed using the program *DIFFaX* (Treacy *et al.*, 1991), which models diffraction from crystals containing one or more phases that may be separated by coherent planar defects including twins and stacking faults. An input file for stacking disorder was obtained from the supplementary material of Malkin *et al*. (Malkin *et al.*, 2012). The model (Fig. 7(a)) consists of (0001) planes of hexagonal ice randomly stacked with (111) planes of cubic ice. The probability of a cubic plane being followed by hexagonal plane is Φ_{ch} and of a hexagonal plane being followed by a cubic plane is Φ_{hc} (Kuhs *et al.*, 2012; Malkin *et al.*, 2015).

To optimize the fits to the diffraction data, *DIFFaX* fitting and background subtraction were embedded in an optimization routine using a bounded, limited-memory BFGS algorithm implemented utilizing the *scipy.optimize.minimize* program in the *SciPy* python library (Oliphant, 2007). The results of this simulation were used to determine the ice crystal's unit cell parameters, stacking probabilities, instrumental broadening, and structure factors. To verify the accuracy of the structure factors, they were also calculated using the explicit equations previously reported (Hansen *et al.,* 2008*b*). As shown in Fig. 9 of (Malkin *et al.*, 2015), calculated ice diffraction profiles show strong variation with the stacking parameters Φ_{ch} and Φ_{hc}, so that values obtained by fit optimization are robust. A single *B*-factor was used for the oxygens and hydrogens in the *DIFFaX* model and was fixed to 1.5, which corresponds to $\langle u^2 \rangle = 0.019$ Å^2. The instrumental broadening was assumed to be purely Lorentzian and parameters *u*, *v*, *w* were optimized to estimate the FWHM broadening as a function of angle.

Fig. 6 (e,f) shows example DIFFaX fits to ice diffraction data from apoferritin and thaumatin crystals. Fig. 7 (b),(c) shows the cubicity parameters – the fraction of all planes that are cubic – deduced from these fits versus temperature and glycerol concentration. Table S1 lists of the number of crystals whose ice diffraction data was fit for each condition. The diffraction patterns and cubicity parameters were highly consistent between crystals at the same temperature and with the same glycerol concentration.

In order to quantitatively estimate ice volume fractions within protein crystals, we required quantitative values of ice diffraction peak intensities. Radial integration of ice peaks and background subtraction to determine these intensities was complicated due to overlap of broadened and

unbroadened peaks from I_{sd}. Diffraction peaks with I_h Miller indices such that $(h-k)/3$ is an integer (Malkin *et al.*, 2012), i.e., the (002), (110), and (112) peaks, are not broadened by stacking disorder, and the other peaks are greatly broadened. The (002) peak is overlapped by the broadened (100) and (101) peaks, the (112) peak is overlapped by the broadened (200) and (201) peaks, while the (110) peak is not overlapped.

To determine the intensities of unbroadened peaks, ice diffraction data were first modelled using *DIFFaX* without including instrumental broadening. Peaks that were not affected by stacking disorder then appeared as sharp spikes (delta functions), and were eliminated from the model's diffraction by interpolating from either side through each peak, leaving only the peaks broadened by stacking disorder. Instrumental broadening was then applied to this model pattern. The sum of the final background and modelled diffraction from the stacking disorder broadened peaks was then subtracted from the measured pattern to determine the intensities of the unbroadened peaks.

S8. Estimating ice fractions in protein crystals

Canonical diffraction equations were used to determine the volume fraction of solvent within each crystal that contributes to the crystalline ice diffraction. The measured diffraction intensities from an initially ice-free protein crystal at room temperature and from the ice that forms within the crystal during and/or after cooling are determined, and relative diffracting volumes of the protein crystal and of ice within the crystal are estimated using the corresponding structure factors for the protein crystal and for the ice, with corrections for unit cell and solvent contractions and solvent expulsion from the unit cell on cooling.

In diffraction data collection from an initially ice-free protein crystal and from the same crystal after ice formation, the X-ray illuminated volume V is the same. The fraction of the illuminated volume that is filled with ice is $f_{ice} = V_{ice}/V$. The fraction of the illuminated volume filled by solvent $f_s = V_s/V$ can be determined by crystallographic analysis of the unit cell. The crystallized solvent fraction is then given by $f_c = f_{ice}/f_s$. Corrections are needed if the ice-free data and solvent volume are determined at room temperature rather than the temperature at which ice forms.

S8.1. Quantifying protein crystal diffraction

A single protein crystal of length $L_{pc}\,(\mathrm{m})$ along the incident beam path, with a unit cell volume $v_{pc}\,(\mathrm{m}^3)$, that is being rotated at an angular velocity $\omega\,(\mathrm{rad/s})$ while being illuminated by an X-ray beam with flux $\Phi\,(\mathrm{photons/s})$ and wavelength $\lambda\,(\mathrm{m})$ produces Bragg peaks whose integrated intensities recorded by the detector $I_{pc,hkl}$ (photons/s) at angles $2\theta_{pc,hkl}$ are given by (Warren, 1990)

$$I_{pc,hkl} = \frac{1}{\omega}\Phi r_e^2 \lambda^3 \frac{L_{pc}}{v_{pc}^2}\left|F_{pc,hkl}\right|^2 \cdot \exp\left[-2B_{pc}\left(\frac{\sin\theta_{pc,hkl}}{\lambda}\right)^2\right] \cdot LP_{pc,hkl} \cdot A^{pc} \cdot A_{pc,hkl}^{air} \cdot A_{pc,hkl}^{detector} \quad . \text{(S5)}$$

Here, $r_e = 2.82\times10^{-15}$ m is the classical electron radius, $\left|F_{p,hkl}\right|$ (electron equivalents) are the crystal's structure factors (including the effects of individual atomic *B* factors), and B_{pc} is a scaling *B*-factor. $LP_{p,hkl}$ is the dimensionless Lorentz-Polarization factor,

$$LP_{pc,hkl} = \frac{1+\alpha\cos^2(2\theta_{pc,hkl})}{(1+\alpha)\sin(2\theta_{pc,hkl})} \quad , \tag{S6}$$

where $\alpha = \cos^2(2\theta_m)$ is the Bragg angle of the monochromator crystal from SI Sec. S2. A^{pc} and $A_{pc,hkl}^{air}$ are the fractional attenuations of the x-ray intensities due to absorption and non-Bragg scattering by the crystal and the air, and respectively. The air absorption model is

$$A_{pc,hkl}^{air} = \exp\left[-\frac{D\mu_{air}\rho_{air}}{\cos 2\theta_{pc,hkl}}\right]. \tag{S7}$$

Here D is the sample-to-detector distance, μ is the x-ray absorption constant at the incident X-ray energy, and ρ is the air density. The product $\mu\rho$ is estimated by *XDS* (whose values agree with those in NIST tables) and is listed in the CORRECT.LP file.

X-ray absorption by the sample is due to absorption by the crystal and the NVH oil. The absorption by the protein crystal is

$$A^{pc}(T) = \exp\left[-\mu_{pc}\rho_{pc}(T)L_{pc}(T)\right]. \tag{S8}$$

The mass absorption coefficient can be assumed constant, and the product of the crystal density and path length varies with cell volume as $\rho L \propto v^{-2/3}$. X-ray absorption increases as the sample contracts because more material is pulled into the beam path. However, assuming a protein crystal density of ~1.3 g/cm^3, a mass absorption coefficient of ~1.2 cm^3/g, and a crystal size of ~400 μm, the 2.5% reduction in apoferritin cell volume observed on cooling to 180 K increases absorption by only ~0.1%, a result that is only slightly modified when absorption by the NVH oil is included.

The Pilatus 6M detector measures photons that are absorbed in a $h = 320\,\mu\text{m}$ thick silicon layer. At $2\theta = 0°$, 75% of the incident photons are absorbed, and at higher diffraction angles the path length through the silicon and the absorption increase. Since only absorbed photons are detected and counted, the measured intensities at small angles are reduced relative to those at large angles. This effect is accounted for using the factor

$$A_{pc,hkl}^{detector} = 1-\exp\left[-\frac{h\mu_{Si}\rho_{Si}}{\cos 2\theta_{pc,hkl}}\right] \quad . \tag{S9}$$

The diffraction equation (S7) can be rewritten as

$$\ln\left[\omega\frac{I_{pc,hkl}}{LP_{pc,hkl}\cdot A_{pc,hkl}^{air}\cdot A_{pc,hkl}^{detector}\left|F_{pc,hkl}\right|^2}\right]=\ln\left[\Phi r_e^2\lambda^3\frac{L_{pc}}{v_{pc}^2}A^{pc}\right]-2B_{pc}\left(\frac{\sin\theta_{pc,hkl}}{\lambda}\right)^2. \qquad \text{(S10)}$$

The slope of a linear fit to a plot of the left side of Eq. S10 versus $2(\sin\theta/\lambda)^2$ can then be used to determine the scaling *B* factor, and the exponential of the *y* intercept gives a constant

$$C_{pc}=\Phi r_e^2\lambda^3\frac{L_{pc}}{v_{pc}^2}A^{pc}, \qquad \text{(S11)}$$

determined by the incident flux and the illuminated protein crystal length along the beam path L_{pc}.

In the present experiments, the 10 frames covering 5° of diffraction data collected from each crystal at room temperature were processed using *XDS*, correcting for the Lorentz polarization factor but with other intensity corrections and scalings turned off (by including 'CORRECTIONS=!' in the XDS.INP file) to obtain absolute intensities in the XDS_ASCII.HKL file. The air and detector absorption corrections were applied manually, since the air absorption correction in *XDS* is relative to rather than absolute and since the formula used for detector absorption correction was not clear, to obtain corrected intensity values $I_{pc,hkl}^{obs}$. Using the default *XDS* intensity corrections and scalings changed final ice volume fractions by less than 1%.

The 5° angular range of the room temperature data was not sufficient for structure determination. To determine the structure factors required to normalize the observed intensities and calculate C_{pc}, we used complete room temperature data sets obtained from crystals that were identically prepared (with identical glycerol concentrations) to those in which ice formed, and processed this data and refined models as described in Section S8 above. Hydrogens were added to the starting PDB model using *phenix.reduce*, and initial structure factors were determined from this model using *phenix.fmodel*. For the flat bulk solvent model assumed in *phenix.fmodel*, the k_{sol} parameter was chosen to be the estimated electron density of the crystal soak solutions, and b_{sol} was set to 50 (Fokine & Urzhumtsev, 2002). To obtain the most accurate structure factor estimates, the structure factors were determined directly using the wk1995 tables (Waasmaier & Kirfel, 1995) rather than using an fft method. The resulting structure factors from *phenix.fmodel* were converted from an .mtz file to a human-readable .csv file that could be compared to the measured intensities from XDS_ASCII.HKL using *phenix.mtz.dump*.

Plots of Eq. S10 were then applied to the region between 10 Å, 5 Å, and 4 Å for apoferritin, thaumatin, and lysozyme, respectively, and the resolution where $I/\sigma=2$ to determine a scaling *B* factor and the constant C_{pc}. Beyond the low-resolution cut-off for each protein, the ratio of measured intensities to structure factors tended to become nonlinear.

To assess the sensitivity of the values of C_{pc} to structural model parameters, several different PDB files (3 apoferritin, 3 thaumatin, and 4 lysozyme) were used, and the resulting variations in calculated

C_{pc} values were 2%, 1.5% and 1.2% for apoferritin, thaumatin, and lysozyme, respectively. Variation of the bulk solvent parameters had little effect on C_{pc}.

Finally, to compare ice and protein crystal diffraction for cooled crystals, the room temperature value of C_{pc} must be scaled from room temperature to the temperature at which ice is observed. This scaling was calculated as

$$\begin{aligned} \frac{C_{pc}(T)}{C_{pc}(300\ \mathrm{K})} &= \frac{L_{pc}(T)}{L_{pc}(300\ \mathrm{K})} \frac{v_{pc}^2(300\ \mathrm{K})}{v_{pc}^2(T)} \frac{A^{pc}(T)}{A^{pc}(300\ \mathrm{K})} \\ &\approx 1 + \frac{5}{3}\frac{\Delta v_{pc}}{v_{pc}} \end{aligned}, \qquad \text{(S12)}$$

wher $\frac{V_{ice}}{v_{pc}} = \frac{L_{ice}}{L_{pc}} = \frac{C_{ice}}{C_{pc}} \frac{v_{ice}^2}{v_{pc}^2}$ $v_{exit}(T) = f_{exit}(T)\, v_{solvent}(T)$ $\Delta v_{pc} / v_{pc}$ is the fractional change in unit cell volume on cooling from room temperature to temperature *T*, and is positive if the unit cell contracts. For the temperatures and protein crystals studied here, the scale factor Eq. S12 is of order 1.05, again a small correction.

S8.2. Quantifying ice diffraction

For a powder sample of ice of length L_{ice} (m) that is exposed for a time *t* (s), the azimuthally averaged and radially integrated diffracted intensity recorded in a diffraction ring at a given angle $2\theta_{ice,hkl}$ is (Warren, 1990)

$$\begin{aligned} I_{ice,hkl} &= \frac{t}{4} \Phi r_e^2 \lambda^3 \frac{L_{ice}}{v_{ice}^2} m_{ice,hkl} \left| F_{ice,hkl} \right|^2 \\ &\times \ \exp\left[-2B_{ice} \left(\frac{\sin\theta_{ice,hkl}}{\lambda} \right)^2 \right] \cdot LP_{ice,hkl} \cdot A^{pc} \cdot A^{air}_{ice,hkl} \cdot A^{detector}_{ice,hkl} \end{aligned}, \qquad \text{(S13)}$$

where $m_{ice,hkl}$ is the multiplicity of the diffraction ring, determined by the number of *hkl* values that produce diffraction at the same 2θ, and all other parameters have the same meaning as for a single crystal in Eq. S5. For an area detector with a sample-detector distance *R* and pixel size δ, the azimuthally integrated intensity per unit length of a pixel is

$$\begin{aligned} I_{ice,hkl,pixel} &= \frac{t\delta}{8\pi R \sin\left(2\theta_{ice,hkl}\right)} \Phi r_e^2 \lambda^3 \frac{L_{ice}}{v_{ice}^2} m_{hkl} \left| F_{ice,hkl} \right|^2 \\ &\times \ \exp\left[-2B_{ice} \left(\frac{\sin\theta_{ice,hkl}}{\lambda} \right)^2 \right] \cdot LP_{ice,hkl} A^{pc} \cdot A^{air}_{ice,hkl} \cdot A^{detector}_{ice,hkl} \end{aligned}. \qquad \text{(S14)}$$

This assumes the detector pixels are on a sphere and subtend a fixed solid angle, which corresponds to our data after *pyFAI* integration and correcting for solid angle. For each ice ring (*hkl* value), Eq. S14 can be rewritten as

$$C_{ice} = \Phi r_e^2 \lambda^3 \frac{L_{ice}}{v_{ice}^2} A^{cryst} = \frac{8\pi R \sin\left(2\theta_{ice,hkl}\right)}{t\delta} \frac{I_{hkl}}{LP_{ice,hkl} \cdot A_{ice,hkl}^{air} \cdot A_{ice,hkl}^{detector} \cdot m_{ice,hkl} \cdot \left|F_{ice,hkl}\right|^2} \times \exp\left[2B_{ice}\left(\frac{\sin\theta_{ice,hkl}}{\lambda}\right)^2\right] . \quad \text{(S15)}$$

Values of C_{ice} from the three diffraction rings that were not broadened by stacking disorder (the hexagonal (002), (110), and (112) rings) were averaged to obtain a final estimate for C_{ice}.

S8.3. Quantifying the crystallizable fraction of crystal solvent

The ratio of the ice and protein crystal constants C_{ice} / C_{pc} at the same temperature T determines the ratio of the volume of ice to the volume of ordered unit cells within the crystal, or equivalently, the volume of ice per unit cell,

$$\frac{V_{ice}}{v_{pc}} = \frac{L_{ice}}{L_{pc}} = \frac{C_{ice}}{C_{pc}} \frac{v_{ice}^2}{v_{pc}^2} . \quad \text{(S16)}$$

Here lower-case v denotes volumes directly associated with the unit cell, determined by crystallography, and an upper case V denotes a volume per unit cell at temperature *T*, for a quantity that can be present both inside and outside the unit cell.

Studies of water at interfaces and of nanoconfined water indicate that there is a layer of interfacial water that is strongly perturbed, and this perturbation should inhibit crystallization. To determine the fraction of a protein crystal's solvent that forms ice, the fraction that cannot be crystallized, and the thickness of this interfacial layer requires careful accounting of both the ice and solvent, both of which can be located within unit cells and within disordered regions of the crystal where solvent exiting the unit cell during cooling accumulates.

The primary quantities of interest are the fraction of the solvent cavity volume within the protein crystal's unit cells that is occupied by ice,

$$f_{cavity,ice} = \frac{V_{ice}^{uc}}{v_{cavity}} , \quad \text{(S17)}$$

and the fraction

$$f_{cavity,solvent\,(\ell)} = 1 - f_{cavity,ice} = \frac{V_{solvent\,(\ell)}^{uc}}{v_{cavity}} \quad \text{(S18)}$$

that is occupied by uncrystallized liquid solvent. The total crystal volume per unit cell is the sum of the volumes of ice, liquid solvent, and protein.

An upper bound on the fraction of the unit cell's solvent cavities occupied by ice can be obtained by assuming that no solvent exits the unit cells on cooling, so that all ice and solvent remain confined within ordered unit cells. In that case, $V_{ice} = V_{ice}^{uc}$, $v_{cavity} = V_{ice} + V_{solvent\,(\ell)}$ so that

$$f_{cavity,ice} = \frac{V_{pc}}{v_{pc}}\left(\frac{1}{1 - v_{protein} / v_{pc}}\right), \tag{S19}$$

where the first term is obtained from Eq. S17 and the second from crystallographic analysis of the unit cell. This is estimate (a) in Table 1.

During cooling, solvent is squeezed out of the unit cell and accumulates at defects and disordered crystal regions, forming pools that are likely larger than the solvent cavities within the unit cell. A second, lower bound on the fraction of the unit cell's solvent cavities occupied by ice can be obtained by assuming that all solvent that exits the unit cell forms ice, and that all liquid solvent is found within the ordered unit cells. In this case, $V_{ice} = V_{ice}^{uc} + V_{ice}^{exit}$, so Eq. S19 becomes

$$\begin{aligned} f_{cavity,ice} &= \frac{V_{ice}^{uc}}{v_{cavity}} = \frac{V_{ice} - V_{ice}^{exit}}{v_{cavity}} \\ &= \frac{V_{ice}}{v_{pc}}\left(\frac{1}{1 - v_{protein} / v_{pc}}\right)\left(1 - \frac{V_{ice}^{exit} / v_{pc}}{V_{ice} / v_{pc}}\right) \end{aligned}. \tag{S20}$$

V_{ice}^{exit} can be approximated by the solvent volume that exits the unit cell, $v_{exit}(T) = f_{exit}(T)\, v_{solvent}(T)$, estimated as described in SI Sec. S5.

Tables 2 and S2 give estimates of the volume fraction of solvent cavities occupied by ice for apoferritin, thaumatin, and lysozyme crystals, based on Eq. S19 (estimate (a)), and on Eq. S20 assuming bulk-like or interface-perturbed solvent contraction (estimates (b) and (c), respectively) as discussed in SI Sec. S5 and given in Table 1.

Ice has a larger volume than the liquid solvent that forms it, but when using the density of supercooled liquid at temperature T this error is at most a few percent. Ice formed in exited solvent, within disordered crystal regions, can draw liquid solvent from within the ordered unit cells for its growth, leading to shrinkage of the unit cell, but such shrinkage was not observed here; only a few of the crystals that formed surface ice exhibited cell shrinkage with time.

S9. Analysis of protein structures from the Protein Data Bank

Unit cell sizes, solvent contents, resolution, and solvent channel sizes were determined from Protein Data Bank entries for 17,148 non-redundant protein structures, excluding small peptides and viral

proteins. 96.8% of entries were determined at temperatures below 180 K; 1.1% were determined at temperatures between 180 and 260 K, and 2.1% were determined at temperatures above 260 K (as indicated by the PDB header files, which can be inaccurate.) Unit cell size, resolution, and solvent content (calculated using the Matthews coefficient (Matthews, 1974; Kantardjieff & Rupp, 2003)) were extracted from the PDB header file. The maximum solvent cavity size was determined using the PDB model coordinates and the program *map_channels* (Juers & Ruffin, 2014) as the diameter of the largest sphere that could be entirely contained within the unit cell's solvent space (whose surface was determined by the Van der Waal's radii of the protein atoms.) The radius of this sphere determines the maximum distance of solvent molecules from the protein surface, the relevant metric for solvent perturbations by that surface. The results of these calculations are shown in Figs. 1, S4, and S5.

For apoferritin, thaumatin, and lysozyme crystals at room temperature, *map_channels* was also used to determine the distribution of solvent distances from the protein surface. The *nc.pdb output file reports the distance between each grid point in a voxelized unit cell and the closest protein surface. The solvent distance distribution, shown in Fig. S6 was obtained by generating a histogram of those distance values belonging to solvent cavity voxels.

S10. Suppression of ice formation in protein crystals

Figs. 3 and S7 show the fraction of apoferritin and thaumatin crystals, respectively, that were cooled to each temperature and remained ice free for at least (a) 3 s and (b) 20 s. Ice nucleation was enormously suppressed relative to rates observed in bulk solutions at all temperatures.

No ice was ever observed above 240 K in either protein, including for glycerol-free crystals. This suggests that 240 K is an upper bound on the freezing temperature of the confined solvent within the glycerol-free crystals (since we cannot be certain that observed ice at lower temperatures did not form in, e.g., larger solvent pockets associated with crystal defects); freezing temperatures decrease to 200-220 K for crystals soaked in 40% w/w glycerol. Fig. 4 compares these freezing temperatures with those of bulk glycerol solutions and of pure water confined in inorganic matrices with different confinement (pore) diameters.

S11. Effects of crystallization salts on ice formation

Any solute that perturbs water's structure and dynamics, including the salts used in crystallization, has cryoprotective abilities (Wang *et al.*, 2016). Apoferritin and thaumatin crystals were crystallized in solutions that, when fully equilibrated with their reservoir solutions, contained ~15% w/v (~1.1 M and 0.5 M) ammonium sulfate and sodium potassium tartrate, respectively. Freezing point suppression at these salt concentrations is at most a few degrees, compared with observed freezing point suppressions in apoferritin and thaumatin crystals of ~30 K. Similarly, measurements on NaCl solutions (Warkentin *et al.*, 2013) suggest that critical cooling rates (the minimum cooling rates required to obtain a glass phase with an ice fraction below ~1% on cooling to 77 K) for these solutions should be ~10^4 K/s. Peak

ice nucleation rates occur between ~230 K and 190 K (Manka *et al.*, 2012), and on cooling at 10^4 K/s to 77 K the sample will spend ~10^{-2} s in this temperature range. In the present experiments apoferritin and thaumatin crystals spent 10^1-10^2 s at these temperatures without formation of detectable ice. Lysozyme crystals were grown in solutions containing only 2.5% w/v NaCl, and exhibited a similar or larger freezing point suppression to apoferritin and thaumatin crystals. Concentrations of salts and other solutes within crystals are generally different (typically somewhat lower) than in crystallization or soaking solutions (Vekilov *et al.*, 1996), but these differences are too small to account for our observations. Consequently, salts present in the crystals provide minimal cryoprotection and cannot explain the dramatic reduction of freezing temperatures and ice formation rates at temperatures between 180 and 260 K.

S12. Connectivity of solvent cavities and ice formation

Among the protein crystals studied here, and also among protein crystals in general, cubic apoferritin is unusual in that much of its solvent is contained in apparently isolated cavities rather than in continuous channels (Fig 3). However, glycerol, iron, and other solutes freely exchange into these cavities at room temperature through 8 hydrophilic channels 6 Å long and 3.4 Å in diameter (Crichton & Declercq, 2010), so they are less disconnected than the static crystallographic structure in Fig. 2 would suggest.

Most studies of nanoconfined water have used nanoporous silica or alumina, in which water is typically confined in a regular matrix of cylindrical channels of nanometer dimensions. Even though the water in adjacent channels is physically separated (except where the matrix has defects or when water extends across the open ends of the channels), experiments suggest that a single ice nucleation event triggers ice formation throughout the matrix (Suzuki, Steinhart *et al.*, 2015). The same may be true in apoferritin, thaumatin, and lysozyme: once ice diffraction is detected, it often saturates to a steady state value in 0.2-0.4 s, except at high (40% v/v) glycerol concentrations, and this steady state value corresponds to conversion of at least a substantial fraction of the crystal's solvent to ice. However, our diffraction measurements have a minimum detectable ice fraction of order 1-2%, and so observed ice may arise from multiple nucleation events.

S13. Cryo- and variable temperature crystallography using cryoprotectant-free protein crystals

For data collection below water's glass transition temperature T_g~136 K, ice formation in most cryoprotectant-free crystals can be outrun by cooling small samples sufficiently rapidly (Warkentin *et al.*, 2006; Pflugrath, 2015). For crystals with smaller unit cells and modest solvent contents (~40% v/v and lower), ice-free diffraction was frequently obtained in the early days of cryocrystallography — before use of penetrating cryoprotectants became standard — by hand-plunging in liquid nitrogen. For crystals with much larger solvent contents and solvent cavities, ice formation on cooling to 77 K can be reliably eliminated by removing external solvent (Warkentin & Thorne, 2009) and using improved

plunge cooling methods that increase cooling rates with liquid nitrogen by one to two orders-of-magnitude (Warkentin *et al.*, 2006).

Far more challenging is data collection at temperatures between 180 and 260 K. Above T_g water molecules develop substantial mobility, and in bulk water or aqueous cryoprotectant solutions at concentrations below ~50-70% v/v ice always forms. Attempts at data collection in this temperature range used crystals soaked in solutions with very large cryoprotectant concentrations (Frauenfelder *et al.*, 1979; Tilton *et al.*, 1992), or else crystals with very low solvent contents and small solvent cavities (Teeter *et al.*, 2001). More recently, careful removal of external solvent has allowed data collection in this temperature range (Warkentin & Thorne, 2010*b*,*a*; Keedy *et al.*, 2015), in some cases without penetrating cryoprotectants. In all of these experiments data collection at each temperature took at least several minutes, and required that crystals remain ice-free during this time. Consequently, they were limited to crystals with modest solvent contents and solvent cavity sizes.

The present results show that cryoprotectant-free data collection can routinely be performed throughout the 180 K-260 K temperature range even with crystals with enormous solvent cavities, by carefully removing or cryoprotecting external solvent, and using intense X-ray beams and fast detectors to record crystal diffraction before ice forms. This dramatically extends the potential scope of variable-temperature crystallographic studies, including to membrane protein crystals with large solvent contents, and to crystals of large complexes having large solvent cavities.

S14. Additional relevant literature references

The present work draws on prior work performed in multiple fields. Space constraints in the main manuscript restricted the number of references we could cite, and in some cases our choice of which references to cite on a given subject was arbitrary. Here we list additional references that informed our work.

Biomolecular cryocrystallography – methods: (Rodgers, 1994; Garman & Schneider, 1997; Garman, 2003; Pflugrath, 2015; Rupp, 2009; Warkentin & Thorne, 2009)

Crystal disorder and degradation of diffraction due to cryocooling: (Kriminski *et al.*, 2002; Juers & Matthews, 2001, 2004*a*,*b*; Alcorn & Juers, 2010; Warkentin *et al.*, 2006; Warkentin & Thorne, 2009)

Temperature-dependent X-ray crystallography of proteins between room temperature and ~180 K: (Frauenfelder *et al.*, 1979, 1987; Douzou *et al.*, 1975; Tilton *et al.*, 1992; Teeter *et al.*, 2001; Warkentin & Thorne, 2010*a*; Warkentin *et al.*, 2012; Keedy *et al.*, 2014, 2015)

Conformational changes in proteins due to cryocooling: (Keedy *et al.*, 2014; Fraser *et al.*, 2011; Atakisi *et al.*, 2018; Halle, 2004; Fraser *et al.*, 2009)

The protein-solvent glass or dynamical transition: (Tilton *et al.*, 1992; Ringe & Petsko, 2003; Schirò *et al.*, 2011, 2015; Doster, 2010; Fenimore *et al.*, 2004)

Measurements of ice nucleation rates in pure water: (Huang & Bartell, 1995; Bartell & Chushak, 2003; Riechers *et al.*, 2013; Chukin *et al.*, 2010; Manka *et al.*, 2012; Murray *et al.*, 2010)

Measurements of ice formation in nanoconfined systems: (Suzuki, Steinhart *et al.*, 2015; Yao *et al.*, 2017; Mascotto *et al.*, 2017; Miyatou *et al.*, 2016; Petrov & Furó, 2011; Morishige & Nobuoka, 1997; Morishige & Kawano, 1999; Findenegg *et al.*, 2008; Jähnert *et al.*, 2008; Schreiber *et al.*, 2001; Taschin *et al.*, 2015; Liu *et al.*, 2010; Rault *et al.*, 2003; Schmidt *et al.*, 1995; Hansen *et al.*, 1996; Bartell & Chushak, 2003)

Simulations of ice formation in nanoconfined systems: (Bartell & Chushak, 2003; Moore & Molinero, 2010; Moore *et al.*, 2010; Gonzalez-Solveyra *et al.*, 2011; Li *et al.*, 2013; Espinosa *et al.*, 2014, 2016)

Water and ice in protein crystals: (Weik, 2003; Weik *et al.*, 2005; Persson *et al.*, 2018; Nakasako, 2004)

Water structure and density near interfaces: (Erko *et al.*, 2012; Persson *et al.*, 2018; Svergun *et al.*, 1998; Merzel & Smith, 2002*a*,*b*; Bagchi, 2005; Wang *et al.*, 2016; Ebbinghaus *et al.*, 2007; Lee *et al.*, 2014)

Ice structure and stacking disorder: (Morishige & Uematsu, 2005; Hansen *et al.*, 2008*a*; Malkin *et al.*, 2012, 2015; Moore & Molinero, 2011; Amaya *et al.*, 2017; Kuhs *et al.*, 1987, 2012)

Table S1 Number of apoferritin and thaumatin crystals that developed internal ice following cooling, and whose ice diffraction patterns were fit at each temperature and glycerol concentration using DIFFAX to obtain the cubicity parameter characterizing stacking disorder.

	Apoferritin			Thaumatin	
T	Glycerol Concentration (v/v)				
	0%	10%	20%	0%	10%
100 K	17			7	
180 K	7	6	4	2	3
200 K	5	6	10	4	2
220 K	2	5	12	1	3

Table S2 Estimates of the maximum fraction of the solvent cavity space occupied by ice in apoferritin and thaumatin crystals at 180<T<220 K

Estimate (a) assumes all solvent is confined to unit cells so all ice forms inside unit cells. Estimate (b) assumes that all solvent has bulk thermal expansion behavior so that solvent exits the unit cells, that all exiting solvent forms ice, and that the volume of ice is the same as the volume of supercooled solvent from which it forms. Estimate (c) instead assumes the solvent density in the first hydration layer is that of bulk, room-temperature solvent, and the remaining solvent has bulk thermal expansion behavior. No systematic variation in ice fractions with temperature between 180 K and 220 K was observed, so values represent averages over all crystals.

Fraction of solvent exiting the unit cell	Fraction of internal solvent that forms ice					
	Apoferritin			Thaumatin		Lysozyme
	0% glycerol	10% glycerol	20% glycerol	0% glycerol	10% glycerol	0% glycerol
(a) $f_{exit} = 0$	59% ± 13%	63% ± 18%	35% ± 9%	35% ± 6%	19% ± 7%	17% ± 5%
(b) f_{exit}, bulk	49% ± 13%	58% ± 18%	33% ± 9%	25% ± 6%	12% ± 8%	8% ± 6%
(c) f_{exit}, perturbed	50% ± 13%	58% ± 18%	33% ± 9%	29% ± 6%	13% ± 8%	12% ± 6%

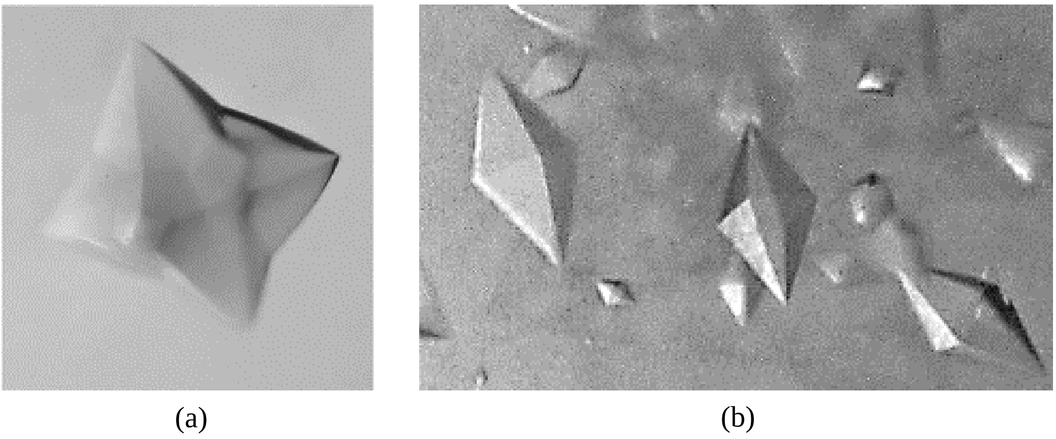

Figure S1 Crystals of (a) cubic apoferritin and (b) tetragonal thaumatin. Crystals are roughly 300 μm in size.

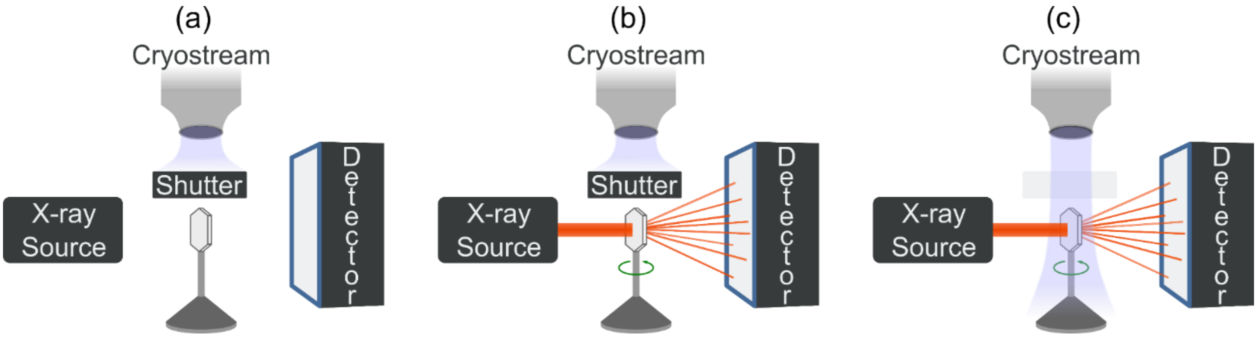

Figure S2 Experimental configuration used in time-resolved X-ray diffraction experiments at the Cornell High-Energy Synchrotron Source (CHESS). (a) sample with its external solvent removed is placed in the X-ray beam path, (b) room temperature data is collected, and (c) the cryostream is unshuttered and data collected while the crystal is cooled.

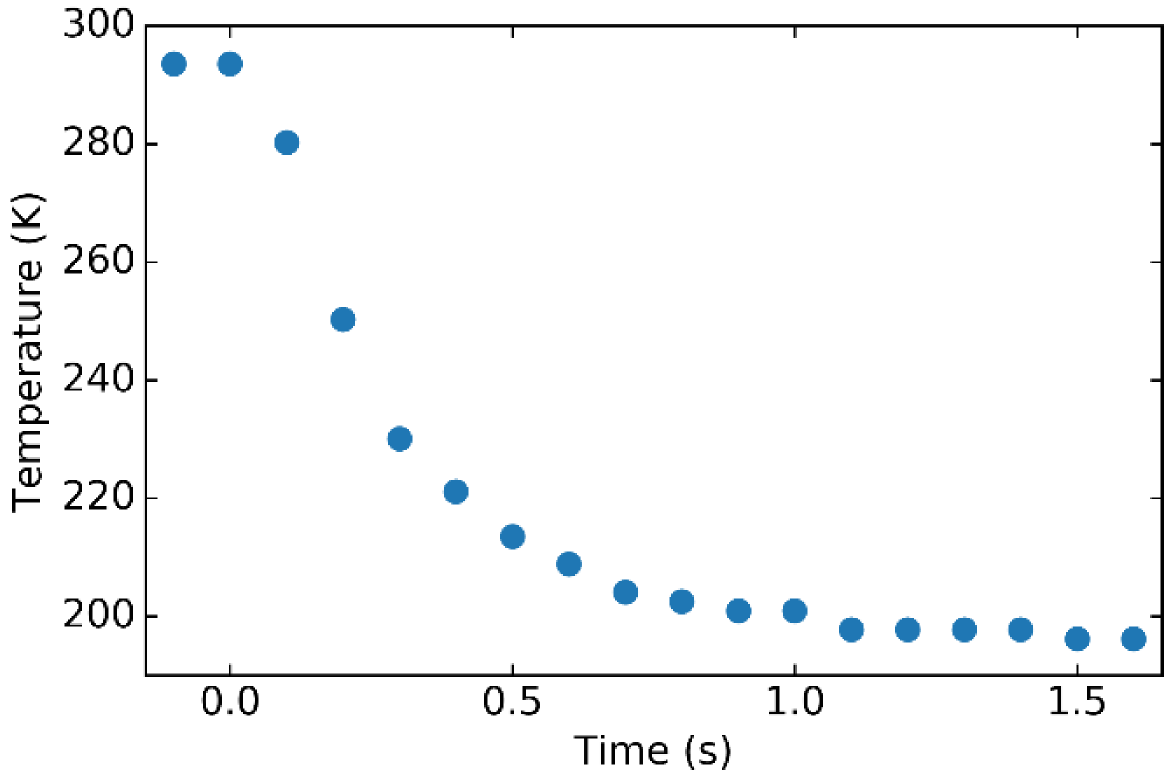

Figure S3 Temperature recorded at the sample position using a 250 μm bead thermocouple when the air blade shutter in Fig. S2 was turned off at t=0. Thermal response times of crystals could be faster or slower, depending on the volume of crystal and surrounding oil.

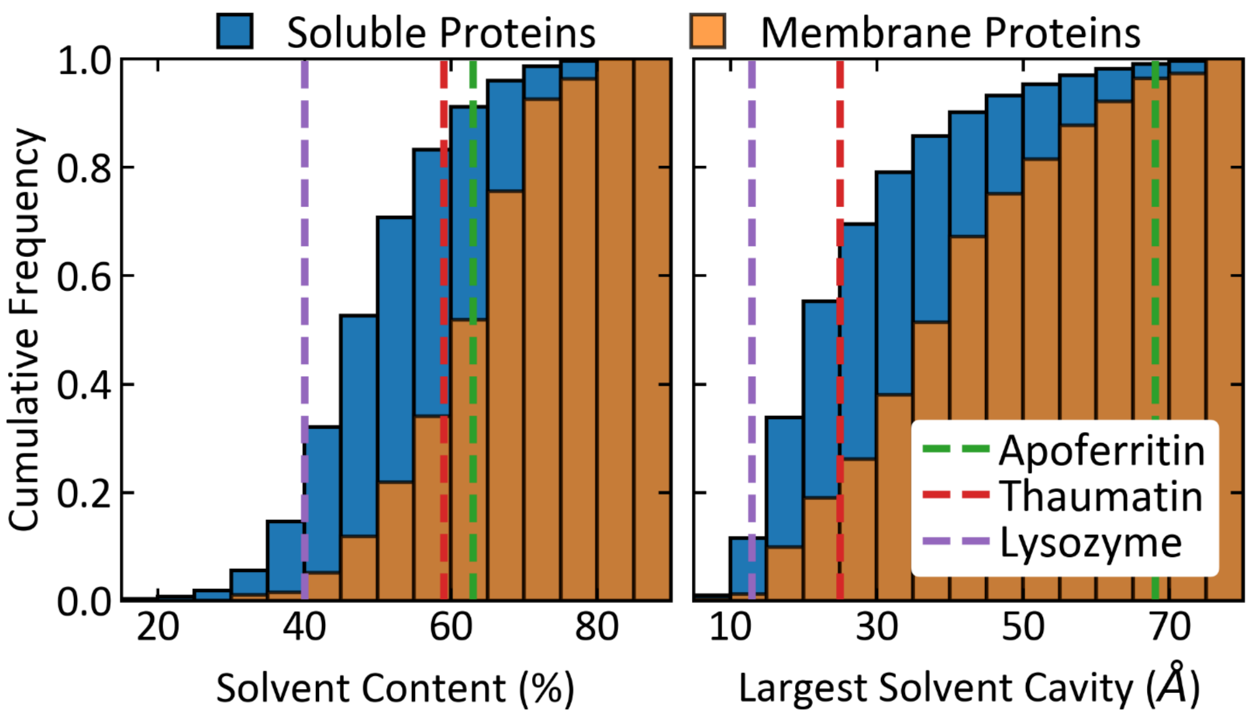

Figure S4 Histograms of the cumulative frequency of (a) the solvent content and (b) the largest solvent cavity obtained from over 17,000 non-redundant structures deposited in the protein data bank (PDB), for soluble proteins (blue) and membrane proteins (orange). The dashed vertical lines indicate the solvent content and largest solvent cavity for lysozyme (purple), thaumatin (red), and apoferritin (green).

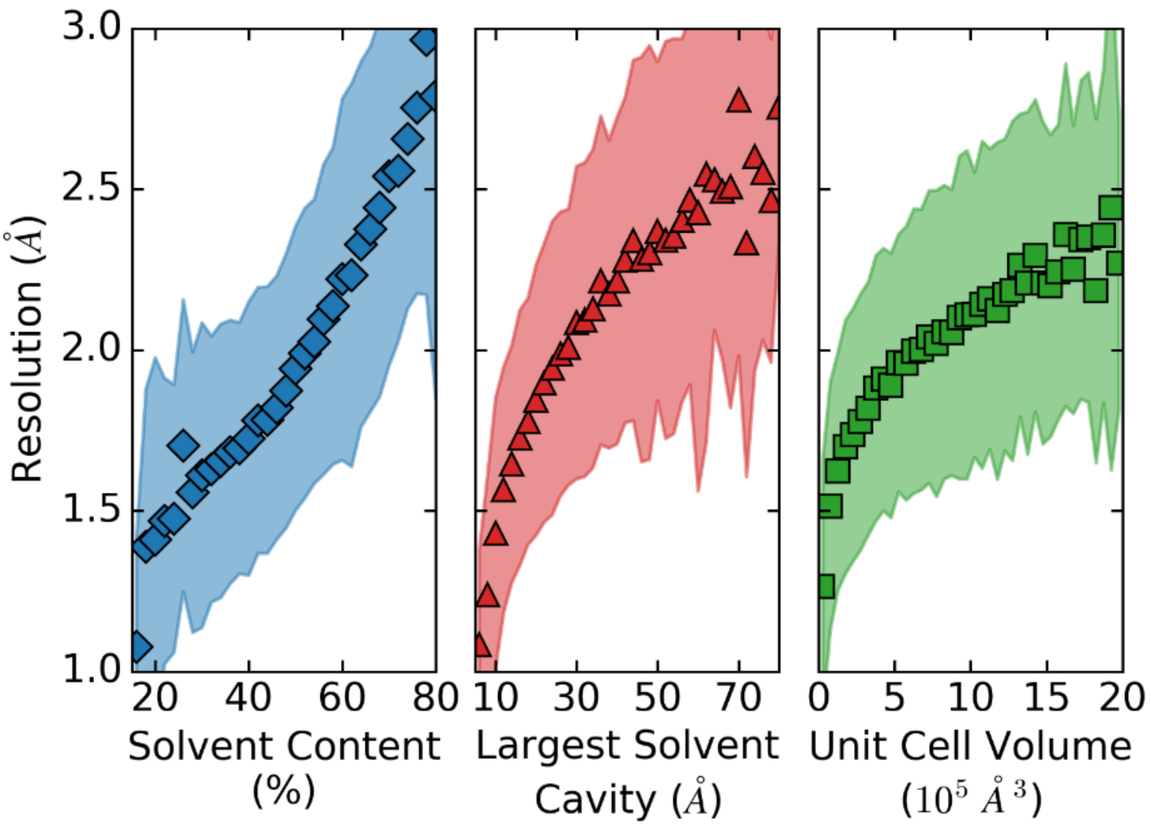

Figure S5 Distribution of diffraction resolution – which determines the highest spatial frequencies in the electron density map of the protein – versus solvent content (% v/v), largest solvent cavity, and unit cell volume for ~17,000 non-redundant cryogenic temperature protein structures obtained from the Protein Data Bank. Symbols represent the mean value over all structures in a given horizontal axis bin, and the boundary of the shaded region shows where the frequency drops to $e^{-1/2}$ of its peak value within each horizontal axis bin.

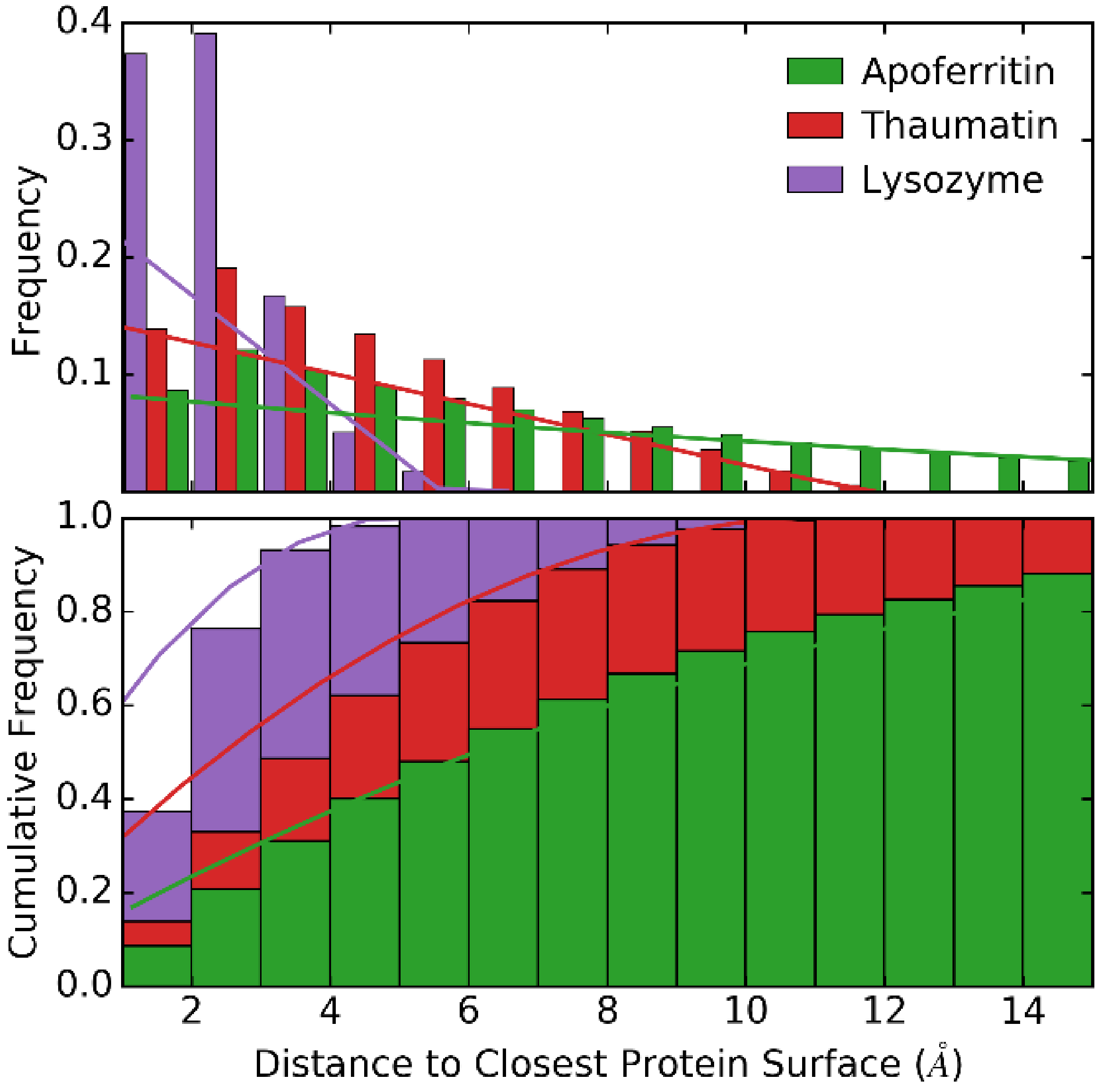

Figure S6 Distribution (top) and cumulative distribution (bottom) of distances from grid locations within the solvent channels to the nearest protein surface in cubic apoferritin, tetragonal thaumatin, and tetragonal lysozyme crystals at room temperature, determined using the results from *map_channels*. Lines represent analytical results for the distance to the closest surface from inside of a spherical shell (apoferritin) or cylindrical shell (thaumatin and lysozyme) of diameter equal to the maximum solvent cavity size in each protein crystal.

Figure S7 Fraction of thaumatin crystals that remained ice free for at least (a) 3 s and (b) 20 s following cooing to each temperature. The numbers in each bar indicate the number of crystals examined under each condition. The smaller fraction of ice-free crystals at 180-220 K relative to apoferritin may be due to differences in crystal handling or to a possible effect on nucleation of the greater connectivity of the solvent spaces within thaumatin crystals.

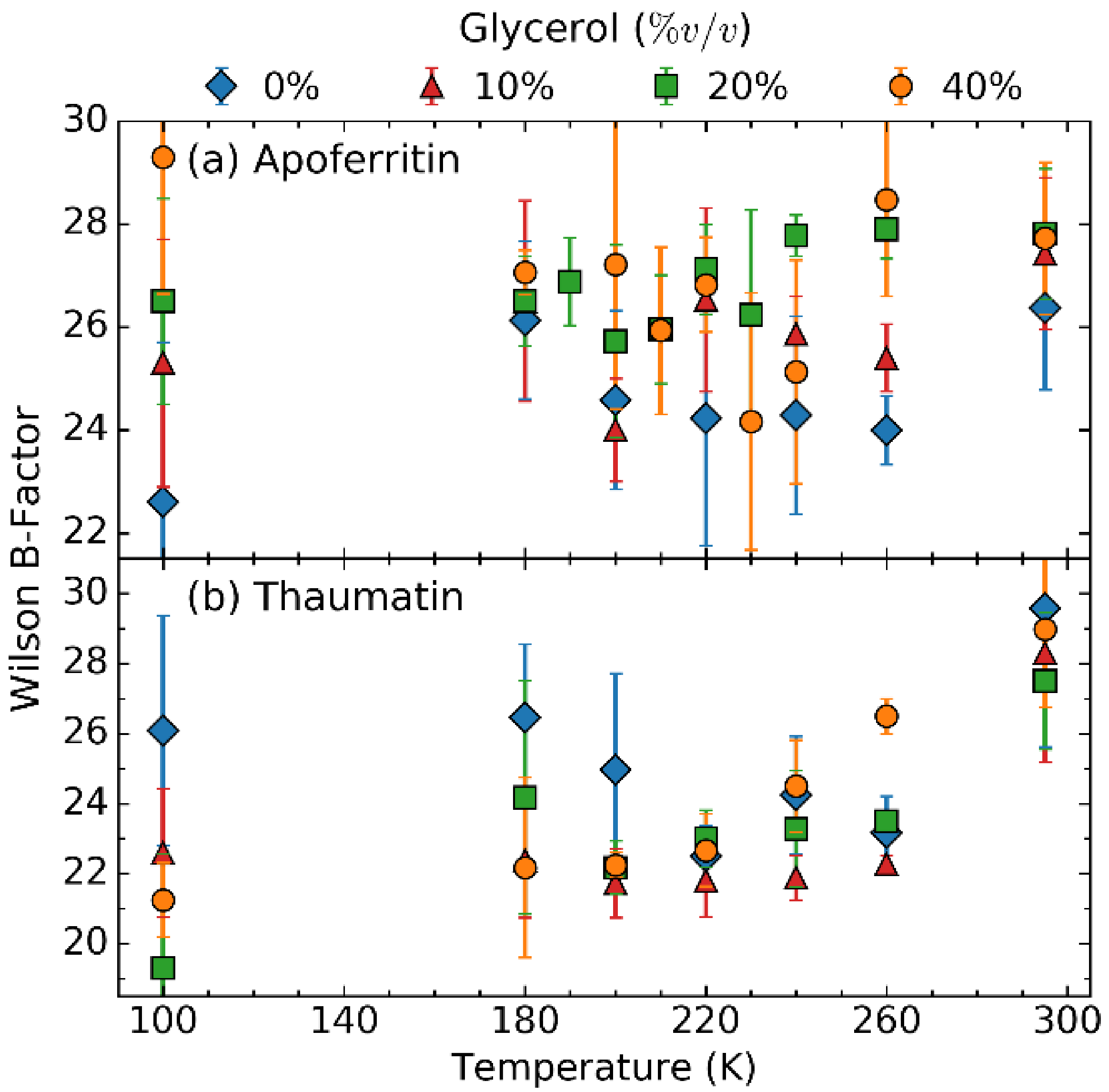

Figure S8 Wilson B factors for (a) apoferritin crystals and (b) thaumatin crystals that cooled without ice formation, versus temperature. For both proteins, cooling to T=100 K produces no clear improvement in B factors relative to 220 K. Significant scatter in values arises from crystal-size-related variations in illuminated volume and background scatter.

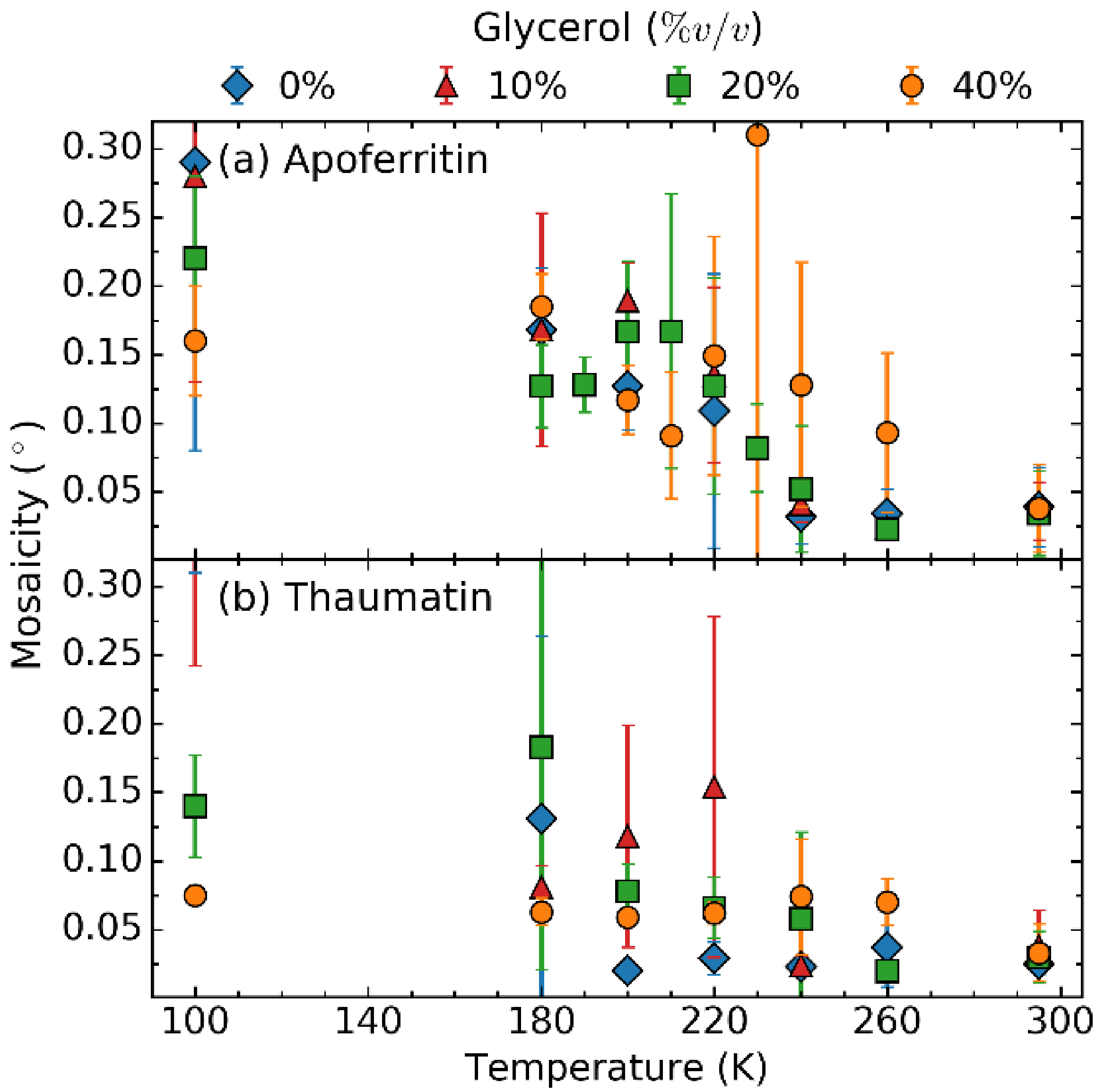

Figure S9 Minimum mosaicities as determined by XDS during the time following cooling that crystals remained ice-free, for (a) apoferritin and (b) thaumatin crystals versus temperature and glycerol concentration. No glycerol-free apoferritin crystals cooled without ice formation to 100 K. XDS-determined mosaicities floored (e.g., for room temperature crystals) at 0.045°. HKL2000-determined mosaicities were roughly 1.4 times larger than XDS mosaicities, but floored at 0.018°, comparable to the incident X-ray beam divergence. Actual room-temperature crystal mosaicities are likely ~0.01° or less for crystals of both proteins.

Movie S1. Animation showing the arrangement of ferritin monomers within the unit cell of cubic apoferritin. The animation was created in *pymol* based on the room temperature structure PDB ID 3F32, using *map_channels* to generate atomic coordinates filling the unit cell.

Movie S2. Animation showing the solvent cavities in cubic apoferritin crystals, based on the room temperature structure with PDB ID 3F32. The program *map_channels* was used to generate a ccp4 map file with map values corresponding to the distance from each grid point to the closest protein surface. In pymol the maps were contoured at 1.6 Å, and the movie.roll function used to create the movies.

Movie S3. Animation showing the protein structure in tetragonal thaumatin crystals. The animation was created in *pymol* based on the room temperature structure PDB ID 3ZEJ, using *map_channels* to generate atomic coordinates filling the unit cell.

Movie S4. Animation showing the solvent cavities in tetragonal thaumatin crystals, based on the room temperature structure with PDB ID 3ZEJ.

Movie S5. Animation showing the protein structure in tetragonal lysozyme crystals at room temperature.

Movie S6. Animation showing the solvent cavities in tetragonal lysozyme crystals at room temperature.

Spreadsheet S1. Refinement statistics for the apoferritin crystals analysed here.

Spreadsheet S2. Refinement statistics for the thaumatin crystals analysed here.

References

Adams, P. D., Afonine, P. V., Bunkóczi, G., Chen, V. B., Davis, I. W., Echols, N., Headd, J. J., Hung, L. W., Kapral, G. J., Grosse-Kunstleve, R. W., McCoy, A. J., Moriarty, N. W., Oeffner, R., Read, R. J., Richardson, D. C., Richardson, J. S., Terwilliger, T. C. & Zwart, P. H. (2010). *Acta Cryst. D*. **66**, 213–221.

Alcorn, T. & Juers, D. H. (2010). *Acta Cryst. D*. **66**, 366–373.

Amaya, A. J., Pathak, H., Modak, V. P., Laksmono, H., Loh, N. D., Sellberg, J. A., Sierra, R. G., McQueen, T. A., Hayes, M. J., Williams, G. J., Messerschmidt, M., Boutet, S., Bogan, M. J., Nilsson, A., Stan, C. A. & Wyslouzil, B. E. (2017). *J. Phys. Chem. Lett.* **8**, 3216–3222.

Ashiotis, G., Deschildre, A., Nawaz, Z., Wright, J. P., Karkoulis, D., Picca, F. E. & Kieffer, J. (2015). *J. Appl. Crystallogr.* **48**, 510–519.

Atakisi, H., Moreau, D. W. & Thorne, R. E. (2018). *Acta Cryst. D*. **74**, 264–278.

Bagchi, B. (2005). *Chem. Rev.* **105**, 3197–3219.

Baker, J. M., Dore, J. C. & Behrens, P. (1997). *J. Phys. Chem. B*. **101**, 6226–6229.

Barbosa, R. D. C. & Barbosa, M. C. (2015). *Phys. A Stat. Mech. Its Appl.* **439**, 48–58.

Bartell, L. S. & Chushak, Y. G. (2003). *Water in Confining Geometries*, Vol. pp. 399–424.

van den Bedem, H., Dhanik, A., Latombe, J. C. & Deacon, A. M. (2009). *Acta Cryst. D*. **65**, 1107–1117.

Brüggeller, P. & Mayer, E. (1980). *Nature*. **288**, 569–571.

Charron, C., Kadri, A., Robert, M., Giege, R. & Lorber, B. (2002). *Acta Cryst. D*. **58**, 2060–2065.

Chen, V. B., Arendall, W. B., Headd, J. J., Keedy, D. A., Immormino, R. M., Kapral, G. J., Murray, L. W., Richardson, J. S. & Richardson, D. C. (2010). *Acta Cryst. D*. **66**, 12–21.

Chukin, V. V., Pavlenko, E. a. & Platonova, a. S. (2010). *Russ. Meteorol. Hydrol.* **35**, 524–529.

Crichton, R. R. & Declercq, J. P. (2010). *Biochim. Biophys. Acta*. **1800**, 706–718.

Datta, S., Biswal, B. K. & Vijayan, M. (2001). *Acta Cryst. D*. **57**, 1162–1167.

Doster, W. (2010). *BBA - Proteins Proteomics*. **1804**, 3–14.

Douzou, P., Hoa, G. H. & Petsko, G. a (1975). *J. Mol. Biol.* **96**, 367–380.

Ebbinghaus, S., Kim, S. J., Heyden, M., Yu, X., Heugen, U., Gruebele, M., Leitner, D. M. & Havenith, M. (2007). *Proc. Natl. Acad. Sci. U. S. A.* **104**, 20749–20752.

Emsley, P., Lohkamp, B., Scott, W. G. & Cowtan, K. (2010). *Acta Cryst. D*. **66**, 486–501.

Erko, M., Wallacher, D., Hoell, a., Hauß, T., Zizak, I. & Paris, O. (2012). *Phys. Chem. Chem. Phys.* **14**, 3852.

Espinosa, J. R., Navarro, C., Sanz, E., Valeriani, C. & Vega, C. (2016). *J. Chem. Phys.* **145**,.

Espinosa, J. R., Sanz, E., Valeriani, C. & Vega, C. (2014). *J. Chem. Phys.* **141**,.

Fahy, G. M. & Wowk, B. (2015). *Cryopreservation and Freeze-Drying Protocols*, Vol. *1257*, edited by W.F. Wolkers & H. Oldenhof, pp. 21–82. New York: Springer.

Fenimore, P. W., Frauenfelder, H., McMahon, B. H. & Young, R. D. (2004). *Proc. Natl. Acad. Sci. U. S. A.* **101**, 14408–14413.

Findenegg, G. H., Jähnert, S., Akcakayiran, D. & Schreiber, A. (2008). *ChemPhysChem*. **9**, 2651–2659.

Fokine, A. & Urzhumtsev, A. (2002). *Acta Cryst. D*. **58**, 1387–1392.

Fraser, J. S., van den Bedem, H., Samelson, A. J., Lang, P. T., Holton, J. M., Echols, N. & Alber, T. (2011). *Proc. Natl. Acad. Sci. U. S. A.* **108**, 16247–16252.

Fraser, J. S., Clarkson, M. W., Degnan, S. C., Erion, R., Kern, D. & Alber, T. (2009). *Nature*. **462**, 669–673.

Frauenfelder, H., Hartmann, H., Karplus, M., Kuntz, I. D., Kuriyan, J., Parak, F., Petsko, G. a, Ringe, D., Tilton, R. F. & Connolly, M. L. (1987). *Biochemistry*. **26**, 254–261.

Frauenfelder, H., Petsko, G. A. & Tsernoglou, D. (1979). *Nature*. **280**, 558–563.

Garman, E. (2003). *Curr. Opin. Struct. Biol.* **13**, 545–551.

Garman, E. F. & Schneider, T. R. (1997). *J. Appl. Crystallogr.* **30**, 211–237.

Halle, B. (2004). *Proc. Natl. Acad. Sci.* **101**, 4793–4798.

Hansen, E. W., Stöcker, M. & Schmidt, R. (1996). *J. Phys. Chem.* **100**, 2195–2200.

Hansen, T. C., Koza, M. M., Lindner, P. & Kuhs, W. F. (2008*a*). *J. Phys. Condens. Matter*. **20**, 285104.

Hansen, T. C., Koza, M. M., Lindner, P. & Kuhs, W. F. (2008*b*). *J. Phys. Condens. Matter*. **20**, 285104.

Hare, D. E. & Sorensen, C. M. (1987). *J. Chem. Phys.* **87**, 4840–4845.

Holten, V. & Anisimov, M. A. (2012). *Sci. Rep.* **2**, 1–7.

Huang, J. & Bartell, L. S. (1995). *J. Phys. Chem.* **99**, 3924–3931.

Hudait, A., Qiu, S., Lupi, L. & Molinero, V. (2016). *Phys. Chem. Chem. Phys.* **18**, 9544–9553.

Jahn, D. A., Wong, J., Bachler, J., Loerting, T. & Giovambattista, N. (2016). *Phys. Chem. Chem. Phys.* **18**, 11042–11057.

Jähnert, S., Vaca Chávez, F., Schaumann, G. E., Schreiber, A., Schönhoff, M. & Findenegg, G. H. (2008). *Phys. Chem. Chem. Phys.* **10**, 6039.

Juers, D. H., Farley, C. A., Saxby, C. P., Cotter, R. A., Cahn, J. K. B., Holton-Burke, R. C., Harrison, K. & Wu, Z. (2018). *Acta Crystallogr. Sect. D Struct. Biol.* **74**, 922–938.

Juers, D. H. & Matthews, B. W. (2001). *J. Mol. Biol.* **311**, 851–862.

Juers, D. H. & Matthews, B. W. (2004*a*). *Q. Rev. Biophys.* **37**, 105–119.

Juers, D. H. & Matthews, B. W. (2004*b*). *Acta Cryst. D.* **60**, 412–421.

Juers, D. H. & Ruffin, J. (2014). *J. Appl. Crystallogr.* **47**, 2105–2108.

Kabsch, W. (2010). *Acta Cryst. D.* **66**, 125–132.

Kantardjieff, K. A. & Rupp, B. (2003). *Protein Sci.* **12**, 1865–1871.

Keedy, D. A., van den Bedem, H., Sivak, D. A., Petsko, G. A., Ringe, D., Wilson, M. A. & Fraser, J. S. (2014). *Structure*. **22**, 1–12.

Keedy, D. A., Kenner, L. R., Warkentin, M., Woldeyes, R. A., Hopkins, J. B., Thompson, M. C., Brewster, A. S., Van Benschoten, A. H., Baxter, E. L., Uervirojnangkoorn, M., McPhillips, S. E., Song, J., Alonso-Mori, R., Holton, J. M., Weis, W. I., Brunger, A. T., Soltis, S. M., Lemke, H., Gonzalez, A., Sauter, N. K., Cohen, A. E., Van Den Bedem, H., Thorne, R. E. & Fraser, J. S. (2015). *Elife*. **4**, 07574.

Kittaka, S., Ishimaru, S., Kuranishi, M., Matsuda, T. & Yamaguchi, T. (2006). *Phys. Chem. Chem. Phys.* **8**, 3223.

Knudsen, E. B., Sørensen, H. O., Wright, J. P., Goret, G. & Kieffer, J. (2013). *J. Appl. Crystallogr.* **46**, 537–539.

Kriminski, S., Caylor, C. L., Nonato, M. C., Finkelstein, K. D. & Thorne, R. E. (2002). *Acta Cryst. D.* **58**,.

Kuffel, A. & Zielkiewicz, J. (2012). *J. Phys. Chem. B.* **116**, 12113–12124.

Kuhs, W., Bliss, D. & Finney, J. (1987). *J. Phys. Colloq.* **48**, 631–636.

Kuhs, W. F., Sippel, C., Falenty, A. & Hansen, T. C. (2012). *Proc. Natl. Acad. Sci. USA*. **109**, 21259–21264.

Lane, L. B. (1925). *Ind. Eng. Chem.* **17**, 924–924.

Lang, P. T., Holton, J. M., Fraser, J. S. & Alber, T. (2014). *Proc. Natl. Acad. Sci. USA*. **111**, 237–242.

Lee, J., Maj, M., Kwak, K. & Cho, M. (2014). *J. Phys. Chem. Lett.* **5**, 3404–3407.

Li, A. J. & Nussinov, R. (1998). *Proteins Struct. Funct. Genet.* **32**, 111–127.

Li, T., Donadio, D. & Galli, G. (2013). *Nat. Commun.* **4**, 1–6.

Liu, D., Zhang, Y., Liu, Y., Wu, J., Chen, C. C., Mou, C. Y. & Chen, S. H. (2008). *J. Phys. Chem. B.* **112**, 4309–4312.

Liu, X. X., Wang, Q., Huang, X. F., Yang, S. H., Li, C. X., Niu, X. J., Shi, Q. F., Sun, G. & Lu, K. Q. (2010). *J. Phys. Chem. B.* **114**, 4145–4150.

Loerting, T., Bauer, M., Kohl, I., Watschinger, K., Winkel, K. & Mayer, E. (2011). *J. Phys. Chem. B.* **115**, 14167–14175.

Malkin, T. L., Murray, B. J., Andrey, V., Anwar, J., Salzmann, C. G., Malkin, T. L., Murray, B. J., Brukhno, A. V, Anwar, J. & Salzmann, C. G. (2012). *Proc. Natl. Acad. Sci.* **109**, 1041–1045.

Malkin, T. L., Murray, B. J., Salzmann, C. G., Molinero, V., Pickering, S. J. & Whale, T. F. (2015). *Phys. Chem. Chem. Phys.* **17**, 60–76.

Manka, A., Pathak, H., Tanimura, S., Wölk, J., Strey, R. & Wyslouzil, B. E. (2012). *Phys. Chem. Chem. Phys.* **14**, 4505.

Mascotto, S., Janke, W. & Valiullin, R. (2017). *J. Phys. Chem. C.* **121**, 23788–23792.

Matthews, B. W. (1974). *J. Mol. Biol.* **82**, 513–526.

Merzel, F. & Smith, J. C. (2002*a*). *Proc. Natl. Acad. Sci. U. S. A.* **99**, 5378–5383.

Merzel, F. & Smith, J. C. (2002*b*). *Acta Crystallogr. Sect. D Biol. Crystallogr.* **58**, 242–249.

Merzel, F. & Smith, J. C. (2005). *J. Chem. Inf. Model.* **45**, 1593–1599.

Miyatou, T., Ohashi, R., Ida, T., Kittaka, S. & Mizuno, M. (2016). *Phys. Chem. Chem. Phys.* **18**, 18555–18562.

Moore, E. B., de la Llave, E., Welke, K., Scherlis, D. a & Molinero, V. (2010). *Phys. Chem. Chem. Phys.* **12**, 4124–4134.

Moore, E. B. & Molinero, V. (2010). *J. Chem. Phys.* **132**, 1–11.

Moore, E. B. & Molinero, V. (2011). *Phys. Chem. Chem. Phys.* **13**, 20008–20016.

Moreau, D. W., Atakisi, H. & Thorne, R. E. *Acta Cryst. D (to Be Submitt.*

Morishige, K. & Kawano, K. (1999). *J. Chem. Phys.* **110**, 4867–4872.

Morishige, K. & Nobuoka, K. (1997). *J. Chem. Phys.* **107**, 6965–6969.

Morishige, K. & Uematsu, H. (2005). *J. Chem. Phys.* **122**,.

Murray, B. J., Broadley, S. L., Wilson, T. W., Bull, S. J., Wills, R. H., Christenson, H. K. & Murray, E. J. (2010). *Phys. Chem. Chem. Phys.* **12**, 10380.

Nakasako, M. (2004). *Philos. Trans. R. Soc. Lond. B. Biol. Sci.* **359**, 1191-1204; discussion 1204-1206.

Oliphant, T. E. (2007). *Comput. Sci. Eng.* **9**, 10–20.

Parsegian, V. A., Rand, R. P. & Rau, D. C. (2000). *Proc. Natl. Acad. Sci. U. S. A.* **97**, 3987–3992.

Persson, F., Söderhjelm, P. & Halle, B. (2018). *J. Chem. Phys.* **148**, 215101.

Petrov, O. & Furó, I. (2011). *Phys. Chem. Chem. Phys.* **13**, 16358.

Pflugrath, J. W. (2015). *Acta Cryst. F*. **71**, 622–642.

Pozharski, E., Weichenberger, C. X. & Rupp, B. (2013). *Acta Cryst. D*. **69**, 150–167.

Rault, J., Neffati, R. & Judeinstein, P. (2003). *Eur. Phys. J. B - Condens. Matter*. **36**, 627–637.

Riechers, B., Wittbracht, F., Hütten, A. & Koop, T. (2013). *Phys. Chem. Chem. Phys.* **15**, 5873.

Ringe, D. & Petsko, G. A. (2003). *Biophys. Chem.* **105**, 667–680.

Rodgers, D. W. (1994). *Structure*. **2**, 1135–1140.

Rupp, B. (2009). Biomolecular Crystallography: Principles, Practice, and Application to Structural Biology. Garland Science.

Saraswathi, N. T., Sankaranarayanan, R. & Vijayan, M. (2002). *Acta Cryst. D*. **58**, 1162–1167.

Sartor, G., Hallbrucker, A. & Mayer, E. (1995). *Biophys. J.* **69**, 2679–2694.

Schirò, G., Caronna, C., Natali, F., Koza, M. M. & Cupane, A. (2011). *J. Phys. Chem. Lett.* **2**, 2275–2279.

Schirò, G., Fichou, Y., Gallat, F.-X., Wood, K., Gabel, F., Moulin, M., Härtlein, M., Heyden, M., Colletier, J.-P., Orecchini, A., Paciaroni, A., Wuttke, J., Tobias, D. J. & Weik, M. (2015). *Nat. Commun.* **6**, 6490.

Schmidt, R., Hansen, E. W., Stoecker, M., Akporiaye, D. & Ellestad, O. H. (1995). *J. Am. Chem. Soc.* **117**, 4049–4056.

Schreiber, A., Ketelsen, I. & Findenegg, G. H. (2001). *Phys. Chem. Chem. Phys.* **3**, 1185–1195.

Segur, J. (1946). *Heat. Vent.* **44**, 86.

Shen, C., Julius, E. F., Tyree, T. J., Moreau, D. W., Atakisi, H. & Thorne, R. E. (2016). *Acta Cryst. D.* **72**, 742–752.

Shimizu, S. & Smith, D. J. (2004). *J. Chem. Phys.* **121**, 1148–1154.

Sinibaldi, R., Ortore, M. G., Spinozzi, F., Carsughi, F., Frielinghaus, H., Cinelli, S., Onori, G. & Mariani, P. (2007). *J. Chem. Phys.* **126**, 235101.

Solveyra, E. G., de la Llave, E., Scherlis, D. a & Molinero, V. (2011). *J. Phys. Chem. B*. **115**, 14196–14204.

Suzuki, Y., Duran, H., Steinhart, M., Kappl, M., Butt, H. J. & Floudas, G. (2015). *Nano Lett.* **15**, 1987–1992.

Suzuki, Y., Steinhart, M., Butt, H. J. & Floudas, G. (2015). *J. Phys. Chem. B*. **119**, 11960–11966.

Svergun, D. I., Richard, S., Koch, M. H., Sayers, Z., Kuprin, S. & Zaccai, G. (1998). *Proc. Natl. Acad. Sci. U. S. A.* **95**, 2267–2272.

Taschin, A., Bartolini, P., Marcelli, A., Righini, R. & Torre, R. (2015). *J. Phys. Condens. Matter*. **27**, 194107.

Teeter, M. M., Yamano, A., Stec, B. & Mohanty, U. (2001). *Proc. Natl. Acad. Sci.* **98**, 11242–11247.

Thorn, A., Parkhurst, J., Emsley, P., Nicholls, R. A., Vollmar, M., Evans, G. & Murshudov, G. N. (2017). *Acta Cryst. D*. **73**, 729–737.

Tilton, R. F., Dewan, J. C. & Petsko, G. A. (1992). *Biochemistry*. **31**, 2469–2481.

Timasheff, S. N. (2002). *Biochemistry*. **41**, 13473–13482.

Toby, B. H. & Von Dreele, R. B. (2013). *J. Appl. Crystallogr.* **46**, 544–549.

Treacy, M. M. J., Newsam, J. M. & Deem, M. W. (1991). *Proc. Roy. Soc. A*. **433**, 499–520.

Tyree, T. J., Dan, R. & Thorne, R. E. (2018). *Acta Cryst. D*. **74**, 471–479.

Vekilov, P. G., Monaco, L. A., Thomas, B. R., Stojanoff, V. & Rosenberger, F. (1996). *Acta Crystallogr. Sect. D Biol. Crystallogr.* **52**, 785–798.

Voss, N. R. & Gerstein, M. (2010). *Nucleic Acids Res.* **38**, 555–562.

Waasmaier, D. & Kirfel, A. (1995). *Acta Cryst. A*. **51**, 416–431.

Wang, Q., Zhao, L., Li, C. & Cao, Z. (2016). *Sci. Rep.* **6**, 26831.

Warkentin, M. A., Sethna, J. P. & Thorne, R. E. (2013). *Phys. Rev. Lett.* **110**, 015703.

Warkentin, M., Badeau, R., Hopkins, J. B., Mulichak, A. M., Keefe, L. J. & Thorne, R. E. (2012). *Acta Crystallogr. Sect. D Biol. Crystallogr.* **68**,.

Warkentin, M., Berejnov, V., Husseini, N. S. & Thorne, R. E. (2006). *J. Appl. Crystallogr.* **39**, 805–811.

Warkentin, M. & Thorne, R. E. (2009). *J. Appl. Crystallogr.* **42**, 944–952.

Warkentin, M. & Thorne, R. E. (2010*a*). *Acta Cryst. D*. **66**, 1092–1100.

Warkentin, M. & Thorne, R. E. (2010*b*). *J. Struct. Funct. Genomics*. **11**, 85–89.

Warren, B. E. (1990). X-ray Diffraction New York: Dover.

Weik, M. (2003). *Eur. Phys. J. B*. **12**, 153–158.

Weik, M., Schreurs, A. M. M., Leiros, H. K. S. K. S., Zaccai, G., Ravelli, R. B. G. & Gros, P. (2005). *J. Synchrotron Radiat.* **12**, 310–317.

Yao, Y., Ruckdeschel, P., Graf, R., Butt, H. J., Retsch, M. & Floudas, G. (2017). *J. Phys. Chem. B*. **121**, 306–313.
